# New Strategy for Cluster‐Based Covalent Organic Framework: Thermally Induced Covalent Crosslinking of Highly Stable Copper Clusters

**DOI:** 10.1002/advs.202507510

**Published:** 2025-07-30

**Authors:** Jian‐Peng Dong, Yue Xu, Ling Yao, Le Wang, Gang Li, Rui Wang, Shuang‐Quan Zang

**Affiliations:** ^1^ Henan Key Laboratory of Crystalline Molecular Functional Materials, and College of Chemistry Zhengzhou University Zhengzhou 450001 P. R. China; ^2^ College of Chemistry and Pharmaceutical Engineering Nanyang Normal University Nanyang 473061 P. R. China

**Keywords:** atomically precise, CO_2_ photoreduction, copper clusters, covalent crosslinking, metal clusters

## Abstract

Copper nanoclusters (Cu NCs) have emerged as a remarkable class of CO_2_ reduction reaction catalysts that are distinguished by their unparalleled reactivity, but effectively modulating the transport pathway of charge carriers between Cu NCs by feasible chemical means is still challenging. Herein, a thermally induced covalent crosslinking strategy is proposed to modulate the fast electron transport pathway formed between clusters. A copper‐sulfur–nitrogen cluster [Cu_4_(SN)_4_] (denoted **Cu_4_SN**) is first synthesized; subsequently, the SN ligands in **Cu_4_SN** are coupled covalently via a thermally induced covalent crosslinking strategy to yield ***CC*–Cu_4_SN**, which exhibits enhanced conductivity and photocarrier transport. As expected, ***CC*–Cu_4_SN** shows a high photocatalytic CO production rate of 29.98 µmol g^−1^ h^−1^ with ≈99.5% selectivity in CO_2_ reduction with H_2_O as sacrificial agents, which is more than 10 times superior to that observed with **Cu_4_SN**. Systematic experiments and density functional theory calculations reveal that the covalent crosslinks between clusters accelerate the dynamic transfer of photoexcited charge carriers, increase the light utilization ability, favor CO_2_ adsorption and ^*^COOH generation, thereby accounting for the increased CO_2_ photoreduction activity. This work presents a novel thermally induced internal covalent crosslinking strategy for synthesizing novel cluster‐based covalent polymers with enhanced stability and catalytic activity.

## Graphical abstract

A thermally induced covalent crosslinking strategy is used to accelerate the photogenerated carrier transport of cluster‐based photocatalysts, the covalent crosslinking between clusters accelerates the dynamic transfer of photoexcited charge carriers, increases the light utilization ability, and favors CO_2_ adsorption as well as ^*^COOH generation, which significantly improved the photocatalytic performance.

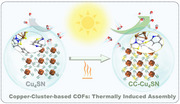

## 1 Introduction

Over the past half century, increasing consumption of fossil fuels has caused a sharp increase in the concentration of CO_2_ in the atmosphere, exacerbating the greenhouse effect and leading to global warming.^[^ ^1^, ^2^ ^]^ Artificial solar‐driven CO_2_ and H_2_O reduction to renewable fuels is one of the most promising approaches for producing carbon‐neutral energy and addressing the global greenhouse effect.^[^ ^3^, ^4^ ^]^ At present, the photocatalysts most reported for the CO_2_ reduction reaction (CO_2_RR) are metal oxides/sulfides,^[^ ^5^ ^]^ metal–organic frameworks (MOFs),^[^ ^6^ ^]^ and conjugated polymers.^[^ ^7^ ^]^ However, the CO_2_RR efficiency and selectivity are still below the thresholds required for practical applications, and the CO_2_RR suffers from low stability, difficulty in controllable fabrication, poor light absorption, fast recombination of photocarriers, and so on.^[^ ^8^ ^]^ In addition, only a very few photocatalysts can realize the overall CO_2_ photocatalytic reaction, which is attributed mainly to the stringent requirements for ideal photocatalysts, including high structural stability, suitable bandgaps, and strong catalytic dynamics.^[^ ^9^, ^10^, ^11^ ^]^ Therefore, seeking more efficient photocatalysts is highly important for overcoming these obstacles.

As an emerging organic‐inorganic crystal material, ligand‐protected Cu NCs have attracted widespread interest because of their well‐defined structure, which is composed of an external organic layer and a metal core.^[^ ^12^, ^13^, ^14^ ^]^ Moreover, the atomically precise crystal structure of Cu NCs provides an ideal model platform for realizing an atomic‐level understanding of catalytic mechanisms.^[^ ^15^, ^16^ ^]^ In addition, compared with most Cu‐based catalysts with unclear atomic surface structures, Cu NCs can be used to purposefully optimize the catalytic performance.^[^ ^17^, ^18^ ^]^ However, to date, Cu‐cluster‐based semiconductors with excellent photocatalytic performance and selectivity for CO_2_ reduction are very rare, which is due mainly to the instability and poor electrical conductivity of assembled Cu NC aggregates employed as heterogeneous photocatalytic semiconductors.^[^ ^19^, ^20^ ^]^ Recently, our group reported a stable crystalline Cu–S–N cluster semiconductor (Cu_6_–NH) and used it as a cluster‐based photocatalyst for efficient overall photocatalytic CO_2_RRs, with H_2_O as an electron donor.^[^ ^21^ ^]^ Owing to the soft and hard sites of the S,N‐type ligand staple clip, these kinds of ligands can produce highly stable metal coordination compounds.^[^ ^22^, ^23^ ^]^ However, the copper cluster molecules are stacked by hydrogen bond interactions, and internal electron transport is limited.^[^ ^17^, ^24^ ^]^ As a result, the poor conductivity always results in the slow transfer of photogenerated charge carriers and hence a limited CO_2_RR rate.^[^ ^9^, ^25^ ^]^ Unfortunately, there are no effective ways to address this problem.

The construction of covalent crosslinks represents one of the strategies most commonly used to generate materials with high electrical conductivity.^[^ ^26^, ^27^ ^]^ Therefore, introducing of covalent crosslinks within clusters may be a viable strategy to increase the inherent electrical conductivity of cluster catalysts, thereby improving photogenerated charge carriers transport and CO_2_ photoreduction efficiency. Owing to strong covalent bonding, cluster‐based covalent organic frameworks (COF) exhibits superior structural stability and internal charge transfer characteristics.^[^ ^28^, ^29^, ^30^, ^31^ ^]^ Conventional covalent assembly typically requires additional organic linkers to connect clusters via chemical reactions between predefined reactive functional groups.^[^ ^32^, ^33^, ^34^, ^35^ ^]^ Given these limitations, developing a novel covalent connection strategy for copper clusters is both highly significant and challenging. Notably, copper‐based materials are promising catalysts for organic bond‐forming reactions.^[^ ^36^, ^37^ ^]^ Cu NCs have been shown to catalyze click chemistry, ketone hydrogenation, and diverse cross‐coupling reactions (C─O, C─N, and C─S).^[^ ^38^ ^]^ In addition, copper‐catalyzed C─S bond formation has been extensively studied for synthesizing sulfur‐containing heterocyclic compounds.^[^ ^39^ ^]^ Based on these advances, we propose a strategy to enhance carrier transport and catalytic performance by constructing covalent interactions between the protective ligands of copper clusters through copper‐catalyzed cross‐coupling reactions.

**Scheme 1:**
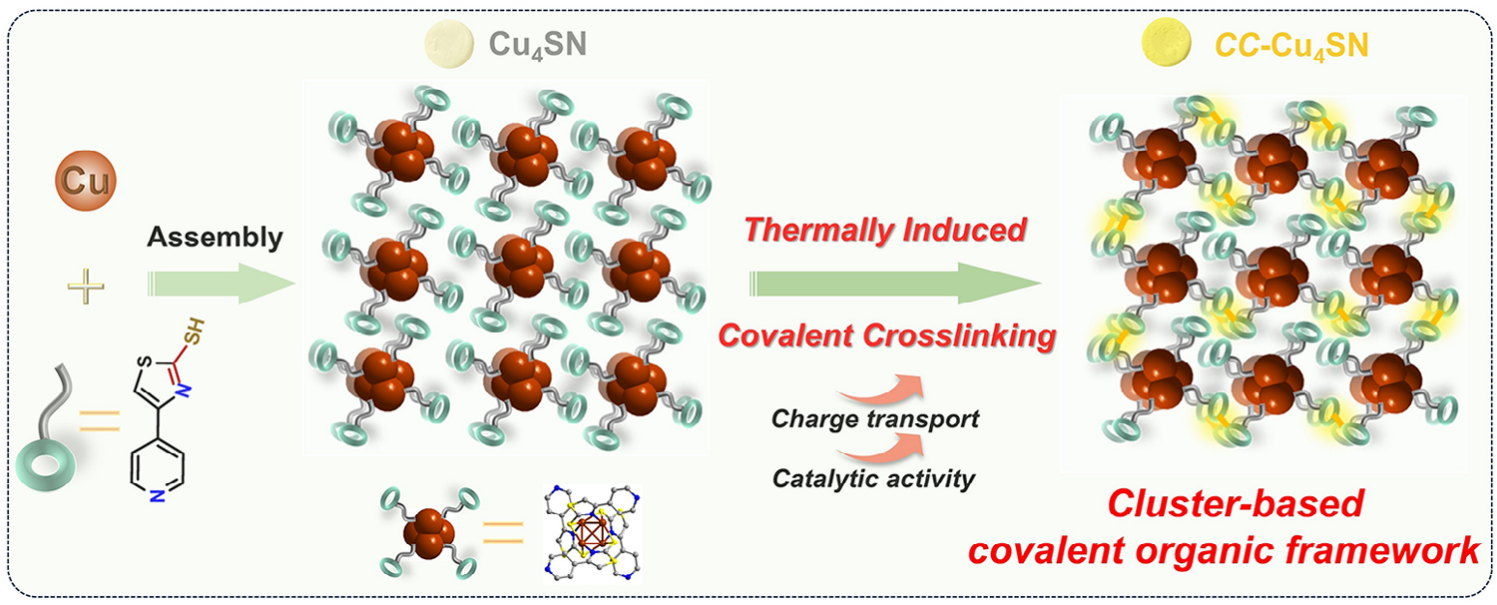
Illustration of the preparation of copper–sulfur–nitrogen cluster‐based photocatalysts through the thermally induced covalent crosslinking strategy.

Herein, we report the synthesis of a novel copper NC, Cu_4_(SN)_4_ (abbreviated as **Cu_4_SN**), protected by an SN ligand (4‐(4‐Pyridinyl) thiazole‐2‐thiol). Subsequently, the SN ligands in **Cu_4_SN** were coupled covalently via a thermally induced covalent crosslinking strategy to yield ***CC*–Cu_4_SN**, which exhibited increased conductivity and photocarrier transport. The thermally induced structural transformations from **Cu_4_SN** to ***CC*–Cu_4_SN** have been systematically characterized via X‐ray photoelectron spectroscopy (XPS), X‐ray absorption fine structure (XAFS), Fourier transform infrared spectroscopy (FT‐IR), solid‐state ^13^C cross‐polarization magic angle spinning nuclear magnetic resonance (^13^C CP/MAS NMR) and thermogravimetric‐differential scanning calorimetry (TGA‐DSC). The results show that a cross‐coupling reaction occurs between the SN ligands of **Cu_4_SN** under thermal induction, and the cluster units are connected by carbon–sulfur bonds. Systematic experiments and density functional theory calculations revealed that the covalent crosslinks between clusters accelerate the dynamic transfer of photoexcited charge carriers, increase the light utilization ability, and favor CO_2_ adsorption and ^*^COOH generation, thereby accounting for the enhanced CO_2_ photoreduction activity (**Scheme** **1**). More importantly, this work is the first example of a thermally induced covalent crosslinking strategy for accelerating the transport of photogenerated charge carriers in cluster‐based photocatalysts, laying the foundation for the development of more functional cluster‐based catalysts through thermally induced covalent crosslinking strategies.

## 2 Results and Discussion

**Figure 1:**
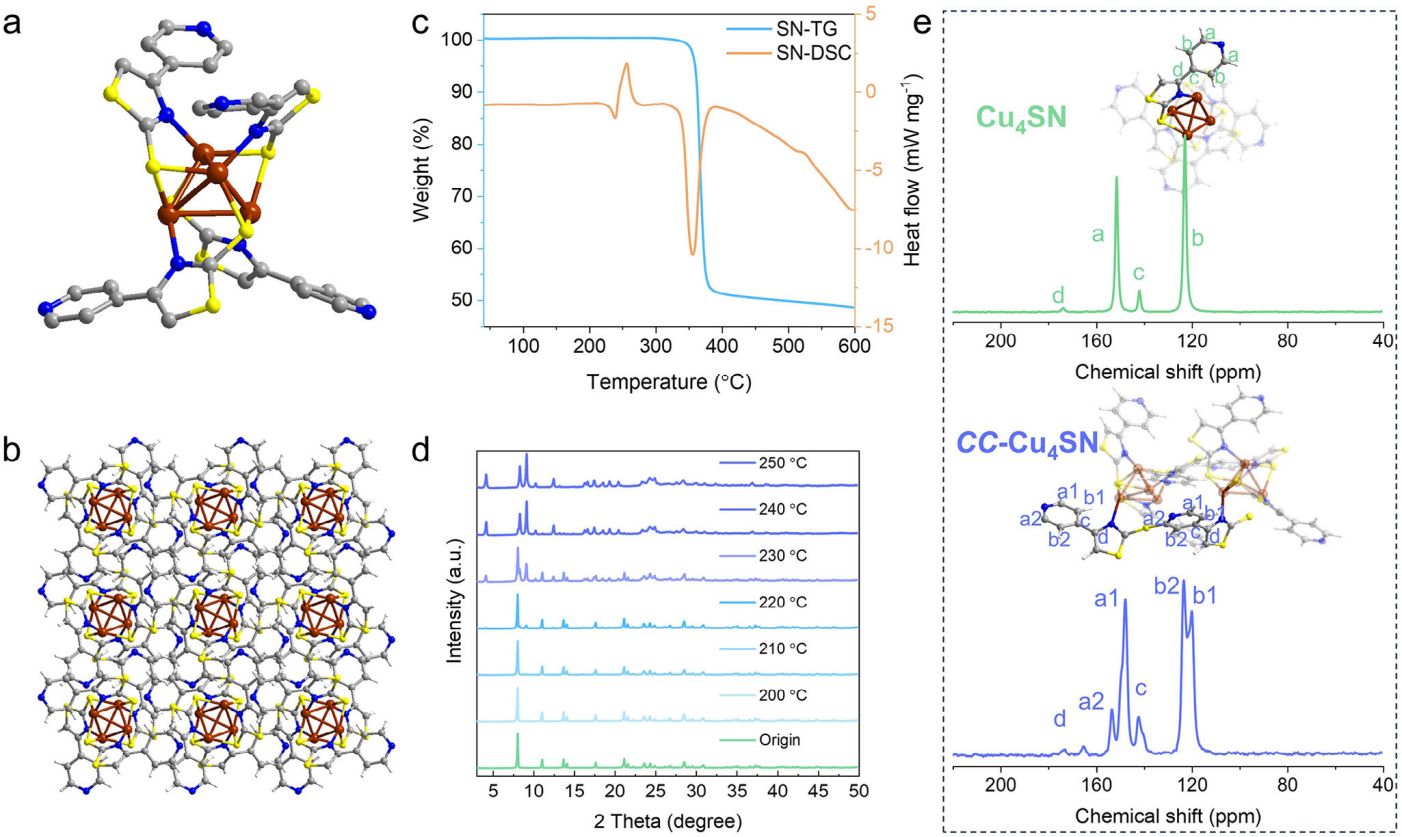
a) Total structures of the **Cu_4_SN** cluster. Color codes: brown = Cu; yellow = S; gray = C; blue = N; white = H. b) Stacking structure of **Cu_4_SN**. c) TG and DSC profile of **Cu_4_SN**. d) PXRD patterns of **Cu_4_SN** treated at different temperatures. e) Schematic model of the structure and ^13^C CP/MAS spectra of **Cu_4_SN** and ***CC*–Cu_4_SN**.

**Cu_4_SN** was prepared by reacting Cu(CH_3_CN)_4_PF_6_ with the SN ligand in a mixed solvent of acetonitrile and methanol; the details are given in the Supporting Information. Single‐crystal X‐ray diffraction (SCXRD) analysis revealed that **Cu_4_SN** crystallized in the tetragonal *I4_1_/a* space group, and the crystallographic information is listed in Table S1 (Supporting Information). As shown in **Figure** **1**a, the inner core is a tetrahedral structure formed by four Cu atoms, every Cu atom is coordinated by two sulfur atoms and a nitrogen atom from three SN ligands, and each SN ligand ligates three Cu atoms. In the tetrahedral Cu_4_ building blocks, the Cu–Cu distances are in the range of 2.716–2.881 Å (for detailed bond lengths, see Table S2, Supporting Information), indicating the presence of weak cuprophilic interactions. Moreover, the Cu─S and Cu─N bond distances are 2.255–2.280 and 1.997 Å, respectively, which agree with the values reported for Cu(I) nanoclusters.^[^ ^40^, ^41^ ^]^ Besides, many hydrogen bond interactions exist between the SN ligands, giving rise to the formation of the 3D network of **Cu_4_SN** (Figure 1b). Furthermore, the phase purity of **Cu_4_SN** was confirmed by powder X‐ray diffraction (PXRD) (Figure S1, Supporting Information) and elemental analysis (EA) (Table S3, Supporting Information).

Thermogravimetric analysis/differential scanning calorimetry coupled with mass spectrometry (TGA/DSC‐MS) was first performed to investigate the thermal degradation characteristics of **Cu_4_SN**. As shown in Figure 1c, no obvious weight loss was detected while heating from RT to 340 °C, which confirms the absence of guest molecules in **Cu_4_SN**. When the temperature is increased from 340 to 380 °C, TG shows a weight loss accompanying the departure of species detected by MS (Figure S2 and Table S4, Supporting Information), and these fragmentation signals are generated by the decomposition of SN ligands. The DSC results revealed that **Cu_4_SN** experienced obvious continuous exothermic and endothermic processes from 220 to 270 °C during the gradual heating process, which indicated that a phase transition occurred in this temperature range.^[^ ^42^ ^]^ The TGA results revealed no significant weight loss or molecular fragment mass spectrum signals in the 220–270 °C range, indicating that **Cu_4_SN** does not decompose at this temperature. However, the distinct endothermic and exothermic processes observed in the DSC results suggest that **Cu_4_SN** undergoes a structural transformation within this temperature range, which inevitably involves the breaking and formation of chemical bonds. There have been numerous reports on the activation of the C─H bond on the pyridinyl ring catalyzed by copper to form new chemical bonds, such as the C─S bond,^[^ ^43^, ^44^ ^]^ and **Cu_4_SN** possesses the elements needed for this reaction, including the copper catalyst (copper core), sulfur (thiolate group on the ligand), and the pyridine ring on the SN ligand. Therefore, we speculate that **Cu_4_SN** undergoes a self‐catalytic reaction under the combined effect of the catalytic action of the copper metal core and the thermal effect, which is also in line with our initial structural design. To further investigate this change, we first subjected **Cu_4_SN** to thermal treatment at different temperatures and obtained its powder X‐ray diffraction (PXRD) patterns. As shown in Figure 1d, starting at 220 °C, the PXRD patterns began to change, and at 240 °C, the structural transformation was complete, which corresponds with the results from the DSC curves. After thermal treatment, **Cu_4_SN** transformed into a new stacked structure. To further elucidate the role of the pyridinyl ring of the SN ligand in **Cu_4_SN** during thermally induced self‐catalysis, a **Cu_4_SC** cluster was prepared using the 4‐phenylthiazole‐2‐thiol ligand for control experiments (Figure S3a, Supporting Information). The structure of 4‐phenylthiazole‐2‐thiol is similar to that of SN; however, the pyridinyl ring of SN is replaced by a benzene ring. As shown in Figure S3b (Supporting Information), the PXRD patterns of **Cu_4_SC** and **Cu_4_SN** are identical, indicating that they possess the same unit cell parameters and identical stacked structures. The TG‒DSC profile of **Cu_4_SC** is depicted in Figure S3c (Supporting Information), and the results show that **Cu_4_SC** remains unchanged up to the ligand decomposition temperature. We also selected 240 °C for the thermal treatment of **Cu_4_SC**, as shown in Figure S3d (Supporting Information), where no change was observed in the PXRD patterns before and after the thermal treatment of **Cu_4_SC**. The only difference between **Cu_4_SN** and **Cu_4_SC** is the presence of the pyridinyl ring on the ligand of **Cu_4_SN** as opposed to the benzene ring on the ligand of **Cu_4_SC**, which further confirms that the structural transformation of **Cu_4_SN** is due to the presence of the pyridinyl ring. On the basis of the above TGA/DSC and PXRD results and comparative studies with **Cu_4_SC**, we selected 240 °C as the optimal treatment temperature (the temperature at which **Cu_4_SN** completed its structural transformation) and subjected **Cu_4_SN** to thermal treatment to obtain ***CC*–Cu_4_SN**.

We subsequently characterized the posttransformation structure. First, the unit cell parameters and stacked structure of ***CC*–Cu_4_SN** were derived with the Rietveld refinement of the PXRD pattern (Figure S4, Supporting Information), with corresponding unit cell parameters of *a* = 21.35 Å, *b* = 10.88 Å, and *c* = 14.25 Å and *α* = 90°, *β* = 87.84°, and *γ* = 90° in the *P*_1_c_1_ space group (Table S5, Supporting Information). The differential plot shows that the refinement of the PXRD pattern is in good agreement with the experimental pattern (unweighted‐profile *R* factor (*R*_p_), 5.43%; weighted profile *R* factor (*R*_wp_), 3.84%). According to the obtained structural model (Figure 1e), the stacked structure of the **Cu_4_SN** cluster units changed. Covalent bonding may have occurred between two adjacent SN ligands through the formation of C‒S bonds. The dehydrogenation of the pyridine ring of the SN ligand to form carbon–sulfur bonds results in the loss of hydrogen atoms. Therefore, we used gas chromatography to detect the gaseous products of the **Cu_4_SN** sample at 240 °C. As shown in Figure S5 (Supporting Information), the production of hydrogen gas was detected, further confirming that dehydrogenation occurred during the transformation of **Cu_4_SN** to ***CC*–Cu_4_SN**, which is also highly consistent with the hypothesis of C─S bond formation. In addition, to ascertain the exact content of elements in ***CC*–Cu_4_SN**, we carried out inductively coupled plasma–mass spectrometry (ICP‐MS) and EA measurements. As shown in Table S3 (Supporting Information), compared with those of the **Cu_4_SN** precursor, the Cu, N, S, and C contents of ***CC*–Cu_4_SN** remain essentially unchanged, with only a slight decrease in the content of hydrogen, which is consistent with the above results. Since the amount of hydrogen lost is minimal, no distinct weight loss signal is observed in the TGA curve.

On the basis of the above discussion, the formation of covalent crosslinks in ***CC*–Cu_4_SN** was further established by a suite of spectroscopic analyses, including solid‐state ^13^C cross‐polarization magic angle spinning nuclear magnetic resonance (^13^C CP/MAS NMR), Fourier transform infrared spectroscopy (FT‐IR), X‐ray photoelectron spectroscopy (XPS) and synchrotron‐radiation‐based X‐ray absorption fine structure (XAFS). In the ^13^C NMR spectra (Figure 1e; Figure S6, Supporting Information), the characteristic chemical shifts at 122, 142, and 151 ppm were assigned to the carbon atoms of the pyridinyl ring in **Cu_4_SN**. The characteristic peak signals at 123, 142, and 149 ppm for ***CC*–Cu_4_SN** could also be associated with carbon atoms of the pyridinyl ring, indicating that the basic pyridinyl ring structure in ***CC*–Cu_4_SN** was well maintained. Notably, the split peaks at 153 and 119 ppm indicate a change in the chemical environment of the carbon atoms on the pyridinyl ring in ***CC*–Cu_4_SN**. This corresponds well with the formation of C─S bonds on the pyridinyl ring, as suggested by the structural model of ***CC*–Cu_4_SN** (Figure 1e). Furthermore, as shown in Figure S7 (Supporting Information), compared with the FT‐IR spectra of **Cu_4_SN**, the C─H stretching vibrations (3000–3100 cm^−1^) and skeleton vibration of the pyridinyl ring (1000–1100 cm^−1^) clearly underwent a large change after transformation to ***CC*–Cu_4_SN**. In addition, a new peak at 760 cm^−1^ appeared in the spectrum of ***CC*–Cu_4_SN**, which was assigned to the stretching vibration of v(C─S),^[^ ^45^ ^]^ further confirming the formation of new C─S bonds between SN ligands of **Cu_4_SN** clusters through the activation of the C─H bond on the pyridinyl ring, which was catalyzed by the copper metal core.

**Figure 2:**
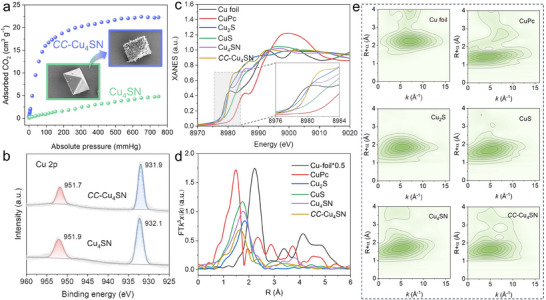
a) CO_2_ adsorption isotherms of **Cu_4_SN** and ***CC–*Cu_4_SN** (inset: SEM images of **Cu_4_SN** and ***CC–*Cu_4_SN**). b) High‐resolution Cu 2p XPS spectra of **Cu_4_SN** and ***CC*–Cu_4_SN**. c) Fourier transform of the Cu *K*‐edge EXAFS of **Cu_4_SN**, ***CC*–Cu_4_SN**, and reference samples Cu foil, CuPc, CuS, and Cu_2_S. The inset is the magnified image. d) Cu K‐edge XANES spectra of **Cu_4_SN**, ***CC*–Cu_4_SN**, and the references (Cu foil, CuPc, CuS, and Cu_2_S). e) Wavelet transforms for the EXAFS signals.

Furthermore, scanning electron microscopy (SEM) and transmission electron microscopy (TEM) were conducted to reveal the morphology of **Cu_4_SN** and ***CC*–Cu_4_SN**. **Cu_4_SN** displays a regular octahedral morphology (**Figure** **2**a; Figure S8, Supporting Information), and the energy‐dispersive X‐ray spectroscopy (EDS) elemental mapping results reveal that C, N, S, and Cu are uniformly distributed in **Cu_4_SN** (Figure S9, Supporting Information). The morphology of ***CC*–Cu_4_SN** was also characterized by SEM and TEM. The EDS elemental mapping results reveal that C, N, S, and Cu are also uniformly distributed in ***CC*–Cu_4_SN** (Figure S9, Supporting Information). Interestingly, ***CC*–Cu_4_SN** maintains a regular octahedral morphology, while its surface transforms into a petal‐like fibre structure (Figure 2a; Figure S8, Supporting Information), which may clearly help to expose more catalytically active sites for CO_2_ adsorption and activation. Therefore, CO_2_ adsorption tests were performed at 298 K, as shown in Figure 2a. The CO_2_ adsorption capacities were 4.74 and 22.25 cm^3^ g^−1^ for **Cu_4_SN** and ***CC*–Cu_4_SN**, respectively, implying that the affinity of ***CC*–Cu_4_SN** for CO_2_ was greater than that of **Cu_4_SN** and the formation of cluster‐based COF.

X‐ray photoelectron spectroscopy (XPS) was used to study the chemical states of **Cu_4_SN** and ***CC*–Cu_4_SN**. The XPS survey data for **Cu_4_SN** and ***CC*–Cu_4_SN** confirm the presence of Cu, N, S, and C (Figure S10, Supporting Information). As shown in Figure 2b, the high‐resolution Cu 2p XPS spectrum of **Cu_4_SN** exhibited a Cu 2p_3/2_ peak at a binding energy of 932.1 eV, which was assigned to Cu^1+^ and was consistent with the absence of a satellite peak and auger line (Figure S11, Supporting Information). Compared with those observed for **Cu_4_SN**, the Cu 2p peaks observed for ***CC*–Cu_4_SN** show a slight negative shift, which can be attributed to the breaking of the Cu–S bond and results in a reduction in the coordination number of the copper atoms.^[^ ^46^ ^]^ Likewise, this phenomenon can be observed from the high‐resolution S 2p and N 1s spectra of ***CC*–Cu_4_SN** (Figure S12, Supporting Information). Notably, after the thermal treatment of **Cu_4_SN**, the relative intensity of the peaks assigned to C─S increased slightly (Figure S13, Supporting Information), indicating that after heat treatment, some new C─S bonds formed, which coincides with the above results.

To further confirm the proposed transformation from **Cu_4_SN** to ***CC*–Cu_4_SN** and study the precise structure of ***CC*–Cu_4_SN**, the oxidation states and local coordination environments of the Cu atoms in **Cu_4_SN** and ***CC*–Cu_4_SN** were investigated by synchrotron‐radiation‐based X‐ray absorption fine structure (XAFS) with Cu foil, CuPc, Cu_2_S and CuS references. As shown in Figure 2c, the Cu K‐edge X‐ray absorption near edge structure (XANES) peaks of **Cu_4_SN** and ***CC*–Cu_4_SN** are located between those of the Cu foil and the Cu_2_S reference and closer to those of Cu_2_S, suggesting the monovalent nature of Cu. Notably, the peak energy of ***CC*–Cu_4_SN** is slightly lower than that of **Cu_4_SN**, suggesting a lower oxidation state of the Cu centre in ***CC*–Cu_4_SN**, which is consistent with the XPS results. Moreover, Fourier transform extended X‐ray absorption fine structure (EXAFS) spectra were obtained to clarify the chemical bonding information for **Cu_4_SN** and ***CC*–Cu_4_SN**. Figure 2d shows the EXAFS spectra in R space, and the dominant peaks in **Cu_4_SN** (1.78) and ***CC*–Cu_4_SN** (1.65) are attributed to the Cu─S path.^[^ ^47^ ^]^ Moreover, compared with that for **Cu_4_SN**, the reduced intensity for ***CC*–Cu_4_SN** indicates a decrease in the coordination number around the Cu centre.^[^ ^48^ ^]^ Additionally, the best fits for **Cu_4_SN** and ***CC*–Cu_4_SN** in R space and K space are shown in Figures S14 and S15 (Supporting Information). The EXAFS fitting results and the fitting parameters are listed in Table S6 (Supporting Information). For **Cu_4_SN**, the results show that the average coordination ratio for Cu─N, Cu─S, and Cu─Cu is 1:2:3, which is in good agreement with the SCXRD results. Moreover, the EXAFS fitting results for ***CC*–Cu_4_SN** indicate that the average coordination ratio for Cu─N, Cu─S and Cu─Cu in ***CC*–Cu_4_SN** is ≈1:1.5:3. The decrease in the average coordination number of Cu─S in ***CC*–Cu_4_SN** proves that some copper–sulfur bonds are broken, which is consistent with the above XPS and FT‐IR results. Additionally, the average coordination numbers of Cu─N and Cu─Cu are unchanged, indicating that the metal core configuration is consistent with that of **Cu_4_SN** and that coordination between the nitrogen atom and the metal core does not change. EXAFS wavelet transform (WT) analysis (Figure 2e) was subsequently conducted to obtain a visual illustration, and the results further support the above conclusion. In addition, the similar oscillation curves of **Cu_4_SN** and ***CC*–Cu_4_SN** obtained via the EXAFS spectra in k space (Figure S15, Supporting Information) suggest a similar structural configuration of the Cu centre, and the shift in the position of the wave peak of ***CC*–Cu_4_SN** is attributed to the slightly increased degree of metal node distortion due to the breakage of partial Cu─S bonds.^[^ ^49^ ^]^ According to the structure of ***CC*–Cu_4_SN** (Figure 1e), the average coordination ratio for Cu─N, Cu─S, and Cu─Cu is 1:1.5:3, which coincides with the EXAFS fitting observations, suggesting the validity of the structural model of ***CC*–Cu_4_SN**. Based on these results, we have a clear understanding of the self‐catalysis process and the transition from **Cu_4_SN** to ***CC*–Cu_4_SN**. Under heating conditions, the Cu─S bonds in **Cu_4_SN** undergo fragmentation, which is then catalyzed by the copper metal core. The pyridine rings on the adjacent SN ligands form covalent connections by creating C–S bonds, which simultaneously undergo a transformation in the stacked structure to form ***CC*–Cu_4_SN**. This process involves the breaking of Cu–S bonds and the formation of new C─S bonds, leading to a change in the molecular architecture and the emergence of a new structural configuration for ***CC*–Cu_4_SN**.

To assess the chemical stability of **Cu_4_SN** and ***CC*–Cu_4_SN**, the samples were exposed to various chemical environments at room temperature, including common solvents such as methanol (MeOH), N, N‐dimethylformamide (DMF), acetonitrile (CH_3_CN), trichloromethane (CHCl_3_), ethyl alcohol (EtOH), and water, for 48 h. As shown in Figure S16 (Supporting Information), the unchanged PXRD patterns revealed that ***CC*–Cu_4_SN** maintained its crystalline structure, which indicates the high chemical stability of ***CC*–Cu_4_SN**. However, for **Cu_4_SN**, the PXRD patterns changed slightly to different degrees after incubation with MeOH, DMF, CH_3_CN, and EtOH. Moreover, there was no obvious change in the PXRD patterns of the **Cu_4_SN** samples incubated with CHCl_3_ or water. Moreover, **Cu_4_SN** and ***CC*–Cu_4_SN** were soaked in 2 m NaOH for 2 days, and the PXRD patterns did not change significantly, indicating the excellent alkali resistance of **Cu_4_SN** and ***CC*–Cu_4_SN**. Nevertheless, ***CC*–Cu_4_SN** exhibited excellent acid resistance, and no changes in crystallinity were observed, even after immersion in a 0.5 m HCl aqueous solution for two days. In contrast, **Cu_4_SN** exhibited relatively poor acid resistance, and the PXRD pattern changed noticeably after immersion in a 0.5 m HCl solution (Figure S16, Supporting Information). The above results show that the stability of ***CC*–Cu_4_SN** is obviously much better than that of **Cu_4_SN** and that the formation of covalent connections significantly increases the stability of the **Cu_4_SN** species.

**Figure 3:**
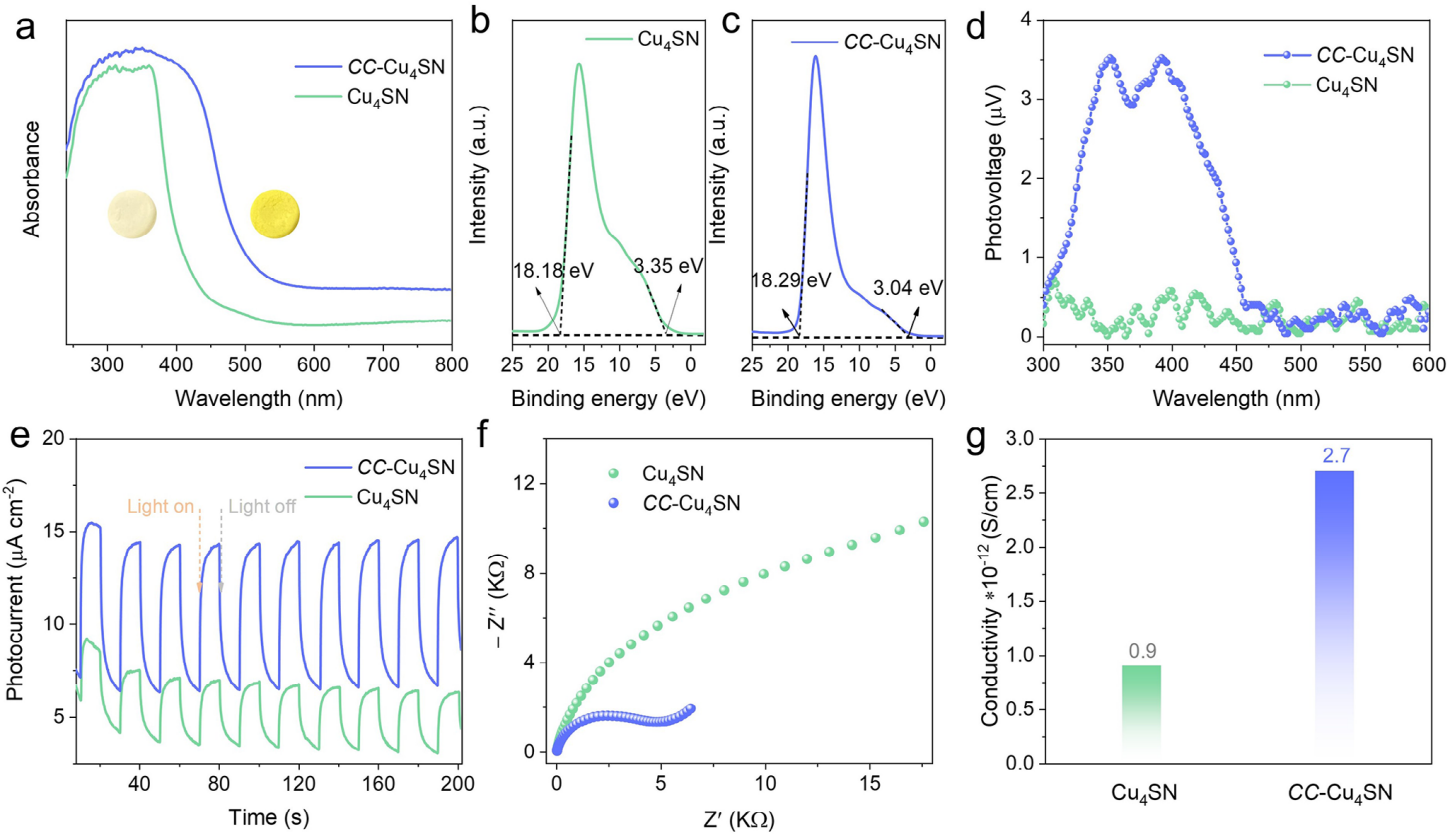
a) Solid‐state UV–vis diffuses reflectance spectra of **Cu_4_SN** and ***CC*–Cu_4_SN**. UPS spectrum of **Cu_4_SN** b) and ***CC*–Cu_4_SN** c). d) Surface photovoltage spectra of **Cu_4_SN** and ***CC*–Cu_4_SN**. e) Transient photocurrent response of **Cu_4_SN** and ***CC*–Cu_4_SN**. f) Electrochemical impedance spectra (EIS) of **Cu_4_SN** and ***CC–*Cu_4_SN**. g) Conductivity of **Cu_4_SN** and ***CC*–Cu_4_SN**.

Interestingly, after thermal treatment, the colour of the sample changed dramatically, from white **Cu_4_SN** to yellow ***CC*–Cu_4_SN**. **Cu_4_SN–T** with a yellow appearance, shows a redshift in absorption. As shown in **Figure** **3**a, **Cu_4_SN** absorbs mainly in the UV region from 200 to 420 nm, with negligible absorption in the visible spectral region, whereas ***CC*–Cu_4_SN** exhibits improved absorption in the visible spectral region (tailing until 550 nm). The optical absorption of **Cu_4_SN** and ***CC*–Cu_4_SN** comes mainly from electronic transitions.^[^ ^50^ ^]^ The enhanced electronic conjugation after the coupling of the SN ligand narrows the *π*→*π* ^*^ gap and increases the rate of electron transfer, which may be the reason for the redshift in the absorption spectrum of **Cu_4_SN** after coupling with the SN ligand, enhancing its light absorption capability in the visible region.^[^ ^51^ ^]^ The bandgap energies (*E*_g_) of **Cu_4_SN** and ***CC*–Cu_4_SN** were 3.14  and 2.58 eV, respectively, on the basis of the Tauc plots (Figure S17, Supporting Information).^[^ ^52^, ^53^ ^]^ Furthermore, ultraviolet photoelectron spectroscopy (UPS) revealed that the valence band maximum (VBM, vs vacuum) is 6.39 eV for **Cu_4_SN** and 5.97 eV for ***CC*–Cu_4_SN** (Figure 3b,c). Following a subtraction of 4.44 eV for conversion to electrochemical potential energy, the VBM (vs NHE) was 1.95 V for **Cu_4_SN** and 1.53 V for ***CC*–Cu_4_SN**.^[^ ^54^ ^]^

Therefore, the conduction band minimum (CBM, vs NHE) of **Cu_4_SN** and ***CC*–Cu_4_SN** were estimated to be −1.19  and −1.05 V, respectively, on the basis of the equation *E*_g_ = *E*_VB_ − *E*_CB_. Therefore, the CBMs of both **Cu_4_SN** and ***CC*–Cu_4_SN** were more negative than the redox potentials of CO/CO_2_ (−0.48 V vs NHE, pH 7), whereas their VBs were more positive than the redox potential of O_2_/H_2_O (+0.82 V vs NHE, pH 7) (Figure S18, Supporting Information). These values confirm that it is thermodynamically feasible for **Cu_4_SN** and ***CC*–Cu_4_SN** to catalyze both the CO_2_ reduction half‐reaction and the H_2_O oxidation half‐reaction.

To display the charge migration dynamics, first, the steady‐state photoluminescence (PL) properties of **Cu_4_SN** and ***CC*–Cu_4_SN** were characterized. As shown in Figure S19 (Supporting Information), the PL intensity of ***CC*–Cu_4_SN** is significantly greater than that of **Cu_4_SN**, which suggests that the formation of covalent connections reduces the number of nonradiative recombination centres in **Cu_4_SN**.^[^ ^55^ ^]^ Moreover, time‐resolved transient PL (TRPL) spectra were also obtained (Figure S20, Supporting Information), and a longer average lifetime was achieved for ***CC*–Cu_4_SN** (1.11 ns) than for **Cu_4_SN** (0.80 ns), indicating the low charge recombination of ***CC*–Cu_4_SN**. The PL intensity and enhanced lifetime of ***CC*–Cu_4_SN** can be attributed to the confinement effect due to the formation of covalent connections between Cu_4_ clusters, which restricts the nonradiative pathways.^[^ ^56^ ^]^ The surface photovoltage (SPV) intensity is positively correlated with the photoinduced charge separation ability.^[^ ^57^ ^]^ Figure 3d shows that the SPV response of ***CC*–Cu_4_SN** was more evident than that of **Cu_4_SN**, which revealed that the charge from ***CC*–Cu_4_SN** was more easily transferred to the surface to participate in the photocatalysis reaction than that from **Cu_4_SN**. Furthermore, the enhanced photogenerated charge carrier separation was confirmed by electrochemical impedance spectroscopy (EIS) Nyquist plots and transient photocurrent responses. As shown in Figure 3e, ***CC*–Cu_4_SN** exhibited a higher photocurrent density than did **Cu_4_SN**. In addition, ***CC*–Cu_4_SN** afforded a smaller Nyquist plot diameter, indicating a lower charge carrier resistance (Figure 3f).^[^ ^58^ ^]^ More importantly, the above results are also supported by the fact that the conductivity of ***CC*–Cu_4_SN** is 3 times greater than that of the **Cu_4_SN** clusters (Figure 3g). Overall, our thermally induced covalent crosslinking strategy can significantly promote light absorption, structural stability, conductivity, photogenerated charge separation, and CO_2_ adsorption, which are crucial for enhancing the photocatalytic CO_2_ reduction activity.

**Figure 4:**
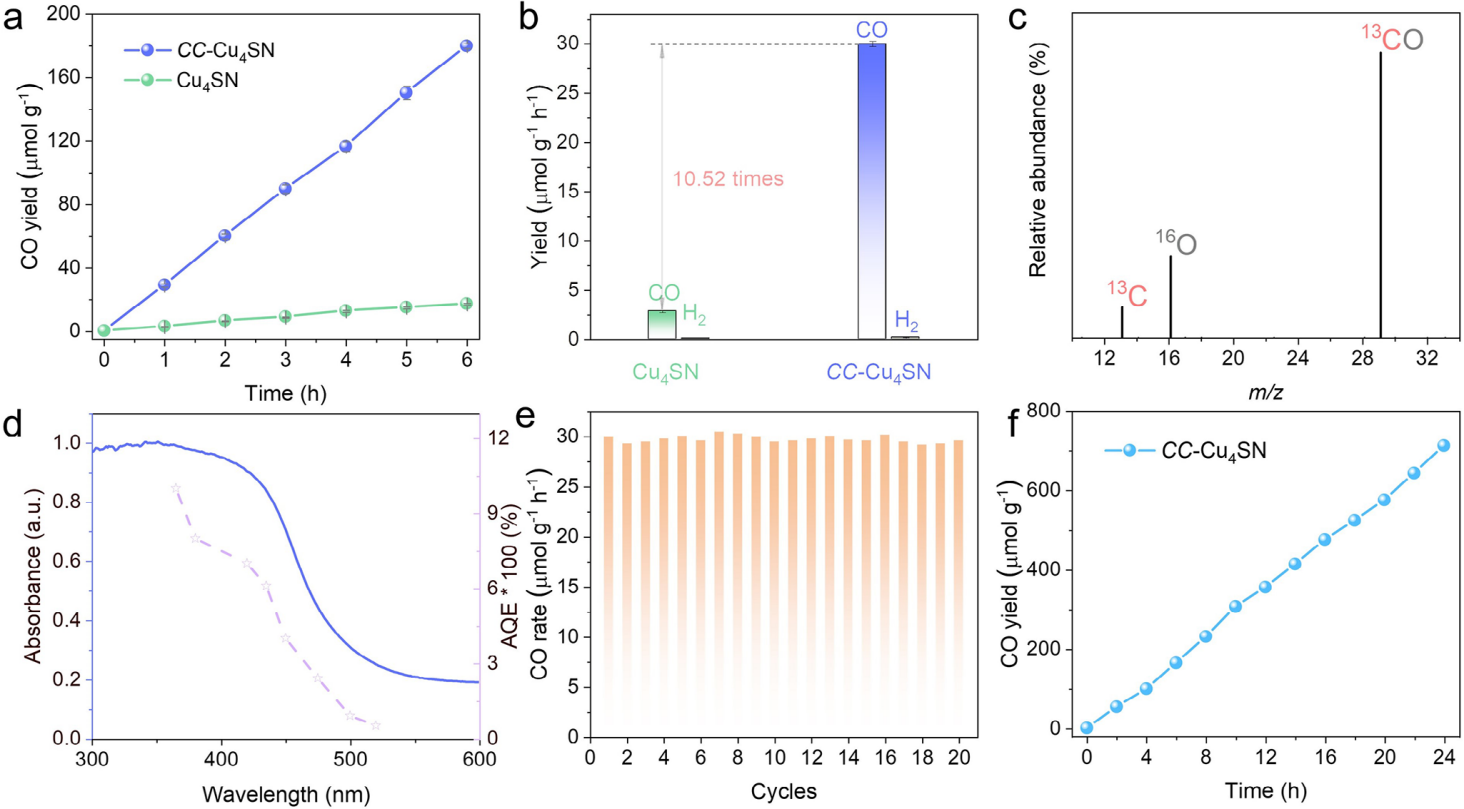
a) Time‐dependent CO_2_–CO photoreduction performances of **Cu_4_SN** and ***CC*–Cu_4_SN**. Error bars derived from triplicate experiments. b) The CO and H_2_ production rate of **Cu_4_SN** and ***CC*–Cu_4_SN**. c) MS spectrum of ^13^CO (*m/z* = 29) produced via the photoreduction of ^13^CO_2_ over ***CC*–Cu_4_SN**. d) Wavelength‐dependent apparent quantum efficiency (AQE) of the CO_2_ reduction to CO over ***CC*–Cu_4_SN**. e) Durability measurements of **Cu_4_SN**–**T** (6 h per cycle, 20 cycles). f) Long‐term photoreduction of CO_2_ over ***CC*–Cu_4_SN**. The system was continuously irradiated by an Xe lamp for 24 h, and gas products were measured at 2 h intervals.

After covalent connections between **Cu_4_SN** clusters are formed, the significantly increased visible light absorption and improved charge transfer dynamics make ***CC*–Cu_4_SN** an ideal candidate for testing how increased photogenerated carrier mobility promotes CO_2_ photoreduction. Therefore, we further explored the CO_2_RR performance of **Cu_4_SN** and ***CC*–Cu_4_SN**. The overall artificial photosynthetic reaction was performed under a pure CO_2_ atmosphere in a CO_2_‐saturated H_2_O solution without the addition of photosensitizers or sacrificial reagents (the detailed photocatalytic process is described in the Methods section). As shown in **Figure** **4**a, the CO yields of **Cu_4_SN** and ***CC*–Cu_4_SN** continue to increase with increasing reaction time. After 6 h, the CO evolution rate of ***CC*–Cu_4_SN** reached ≈179.88 µmol g^−1^, almost 10.52 times higher than that of **Cu_4_SN** (≈17.10 µmol g^−1^). The CO yield of ***CC*–Cu_4_SN** was not only much better than that of **Cu_4_SN** but also better than that of many previously reported photocatalysts under similar conditions (Table S7, Supporting Information). Notably, **Cu_4_SN** and ***CC*–Cu_4_SN** exhibited excellent CO selectivities of 98.28% and 99.50%, respectively (Figure 4b). A small amount of H_2_ was detected, and only CO was detected as a carbon‐based product (Figure S21, Supporting Information); meanwhile, the nuclear magnetic resonance spectra confirmed the absence of liquid carbon‐based products (Figure S22, Supporting Information). The blank experiments over ***CC*–Cu_4_SN** were performed in the absence of light, CO_2_, water, or photocatalyst, and no CO or other carbon‐based chemicals were detected (Figure S23, Supporting Information). In addition, to further verify the origin of CO, the isotope ^13^CO_2_ was used as the substrate for the photocatalytic CO_2_ reduction reaction over ***CC*–Cu_4_SN**. As shown in Figure 4c, the signal peak at *m/z* = 29 (^13^CO) verified that the generated CO originated from CO_2_ reduction. To further evaluate the photocatalytic activity of ***CC*–Cu_4_SN**, an apparent quantum efficiency (AQE) test was conducted under a series of monochromatic light wavelengths (Figure 4d). The AQE trend matched the absorption spectrum of ***CC*–Cu_4_SN**, indicating that the CO_2_ reduction reaction depends mainly on the wavelength of the incident light and is driven by the photocarriers of ***CC*–Cu_4_SN**, which coincides with the CO generation observations.^[^ ^21^ ^]^ Moreover, O_2_ could also be detected, which was attributed to the oxidation of H_2_O by the photogenerated holes. The O_2_ evolution rate is almost half that of CO (Figures S24 and S25, Supporting Information), which is consistent with the electron consumption ratio for CO_2_‐to‐CO conversion (2e^−^) and the water oxidation reaction (4e^−^).

As is well known, the stability of a catalyst is very important. The photocatalytic stability of ***CC*–Cu_4_SN** was studied by using the same catalyst over 20 continuous cycles (120 h) by evacuation and refilling with CO_2_, as shown in Figure 4e, and the photocatalytic activity of ***CC*–Cu_4_SN** was largely maintained. Moreover, no obvious decrease in the CO generation rate was observed after continuous reaction for 24 h (Figure 4f). PXRD, FT‐IR, TEM, EDX, XPS, ICP‒MS, and EA studies were conducted after photocatalysis to characterize the cycled ***CC*–Cu_4_SN** catalyst. The PXRD (Figure S26, Supporting Information) patterns, FT‐IR (Figure S27, Supporting Information) spectra, and ICP‒MS and EA results (Table S8, Supporting Information) showed no detectable changes after the catalysis reactions. Moreover, the TEM and EDX mapping patterns (Figure S28, Supporting Information) confirmed that the structure and morphology of the cycled catalyst were maintained. Additionally, XPS analysis of ***CC*–Cu_4_SN** was performed postcatalysis to exclude the possible photoetching of photocatalysts (Figure S29, Supporting Information), which highlights the robust stability and excellent durability of ***CC*–Cu_4_SN**.

**Figure 5:**
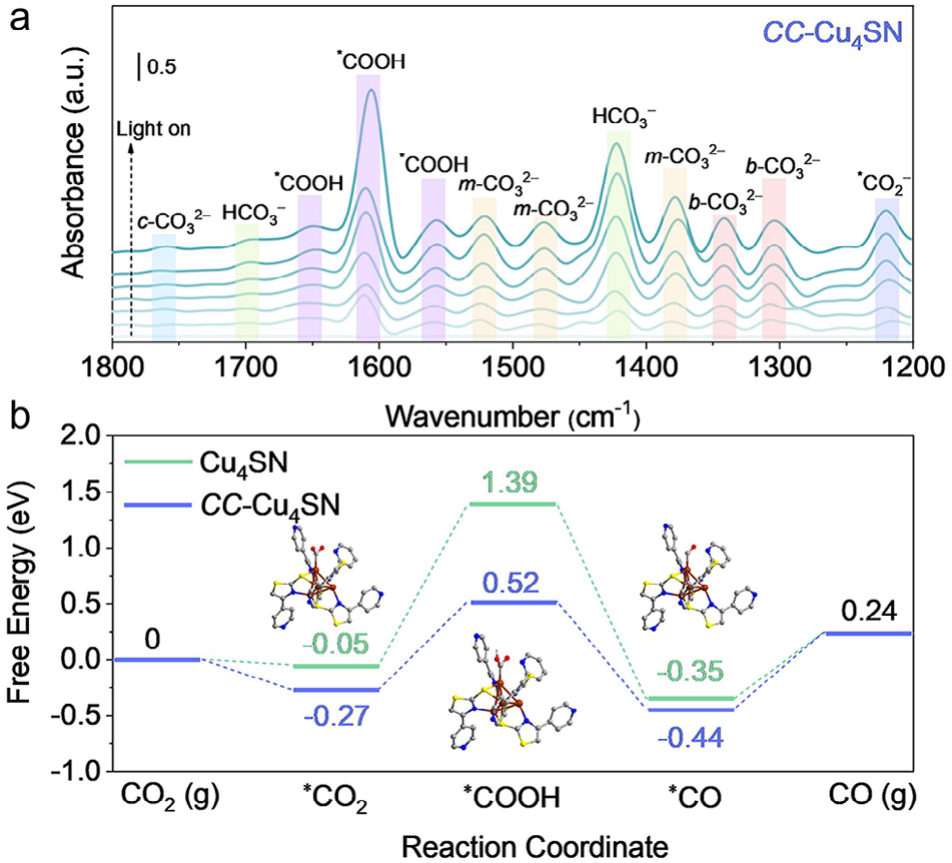
a) In situ DRIFTS spectra at different irradiation times during the CO_2_ photoreduction over ***CC*–Cu_4_SN**. b) Free‐energy diagrams of the CO_2_ photoreduction to CO over **Cu_4_SN** and ***CC*–Cu_4_SN**.

To elucidate the photocatalytic CO_2_ reduction pathway, in situ DRIFTS measurements were performed to detect the reaction intermediates during reduction. **Figure** **5**a shows that the peaks of key intermediates are gradually enhanced with prolonged exposure to light irradiation for ***CC*–Cu_4_SN**. The peaks at 1556, 1605, and 1649 cm^−1^ for ***CC*–Cu_4_SN** could be assigned to the intermediate of the COOH^*^ radical,^[^ ^59^, ^60^ ^]^ which is generally regarded as the crucial intermediate in CO_2_ reduction to CO. In addition, the peak at 1220 cm^−1^ is attributed to the carboxylate group (^*^CO_2_^−^) vibration, whereas the absorption bands at 1420 and 1698 cm^−1^ are indicative of the symmetric stretching of ^*^HCO_3_^−^.^[^ ^61^, ^62^ ^]^ Moreover, the peaks at 1305, 1342 and 1760 cm^−1^ were also detected, which could be attributed to the asymmetric OCO stretching of the bidentate carbonate (*b*‐CO_3_^2−^) and chelating‐bridged carbonate (*c*‐CO_3_^2−^) groups,^[^ ^21^, ^63^ ^]^ while the peaks at 1377, 1478 and 1520 cm^−1^ could be attributed to monodentate carbonate (*m*‐CO_3_^2−^) groups.^[^ ^64^, ^65^ ^]^ These carbonate and bicarbonate peaks indicate that CO_2_ and H_2_O molecules were adsorbed onto the photocatalysts. Notably, except for the peak intensity, no significant difference between **Cu_4_SN** and ***CC*–Cu_4_SN** can be observed. As shown in Figure S30 (Supporting Information), the in‐situ DRIFTS peak locations of **Cu_4_SN** are basically the same as those of ***CC*–Cu_4_SN**; the difference is that the peak intensity of **Cu_4_SN** is much lower, which coincides with the results of the photocatalytic yield tests. On the basis of the above analysis, the reduction mechanism is proposed as follows:

(1)

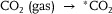



(2)

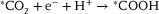



(3)

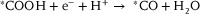



(4)

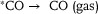




To gain deep insight into the reaction mechanism and further investigate the variation in catalytic performance caused by the cluster covalent linkages, DFT calculations were conducted. As depicted in Figure 5b, the calculated Gibbs free energy for ^*^CO_2_ formation was lower than the initial value, which implies that CO_2_ adsorption on **Cu_4_SN** and ***CC*–Cu_4_SN** was exothermic. In addition, ***CC*–Cu_4_SN** has a greater CO_2_ adsorption ability, which is attributed to it having a smaller energy barrier (−0.27 eV) than **Cu_4_SN** does (0.05 eV). The thermodynamic feasibility of ***CC*–Cu_4_SN** implies its strong affinity toward CO_2_ in comparison with that of **Cu_4_SN**, which is consistent with the above CO_2_ absorption results. Furthermore, the results suggested that the formation of the COOH^*^ intermediate with the highest energy barrier is the rate determining step (RDS) for **Cu_4_SN** (1.44 eV) and ***CC*–Cu_4_SN** (0.79 eV), where the much lower energy barrier of ***CC*–Cu_4_SN** supports the higher catalytic activity of ***CC*–Cu_4_SN** to **Cu_4_SN**, indicating that the presence of covalent linkages between Cu_4_ clusters could help optimize the rate‐limiting step and hence accelerate the photocatalytic CO_2_ reduction performance. The high selectivity for CO generation was subsequently investigated (Figure S31, Supporting Information). For **Cu_4_SN** and ***CC*–Cu_4_SN**, the free energy for ^*^CHO formation was greater than the desorption energy of CO molecules. This implies that ^*^CO desorption from both **Cu_4_SN** and ***CC*–Cu_4_SN** is more favorable than the proton‐coupled electron transfer of ^*^CO to produce ^*^CHO, which explains their high selectivity for the reduction of CO_2_. Moreover, operando XAFS measurements were conducted to probe the dynamic evolution of Cu oxidation states and elucidate their function in the photocatalytic CO_2_ reduction process over ***CC*–Cu_4_SN**. Cu K‐edge XANES spectra were acquired in situ during actual CO_2_RR operation. Under dark conditions within the CO_2_ atmosphere, the Cu K‐edge exhibited a positive shift of ≈0.11 eV relative to the pristine material (Figure S32, Supporting Information). This shift is attributed to spontaneous electron donation from Cu active sites to the C 2p orbital of CO_2_, signifying an increased Cu oxidation state necessary for effective CO_2_ adsorption and activation.^[^ ^40^ ^]^ Upon light irradiation, the Cu K‐edge energy in ***CC*–Cu_4_SN** progressively reverted toward lower values (Figure S32, Supporting Information), demonstrating the partial regeneration of low‐oxidation‐state Cu species through the acceptance of photogenerated electrons, thereby facilitating CO_2_ reduction. Consequently, within this photocatalytic system, copper centers in ***CC*–Cu_4_SN** function as electron acceptors, driving the CO_2_ reduction reaction.

## 3 Conclusion

In summary, we demonstrate that via a novel thermally induced self‐catalysis covalent crosslinking strategy, a stable Cu─S─N cluster‐based COF, ***CC*–Cu_4_SN**, driven by covalent interactions is reported for the first time, which was unambiguously elucidated by ^13^C CP/MAS NMR, PXRD, FT‐IR, XPS, and XAF analyses, revealing that a cross‐coupling reaction occurs between the SN ligands of **Cu_4_SN** clusters under thermal induction and that the cluster units are connected by the formation of carbon–sulfur bonds. ***CC*–Cu_4_SN** could serve as an efficient single‐component photocatalyst that displays a high overall photocatalytic CO_2_RR efficiency with H_2_O under visible light irradiation that is more than 10 times greater than that of **Cu_4_SN**. Systematic experiments and density functional theory calculations revealed that the covalent crosslinks between clusters accelerate the dynamic transfer of photoexcited charge carriers, increase the light utilization ability, and favor CO_2_ adsorption and ^*^COOH generation, thereby accounting for the increased CO_2_ photoreduction activity. Therefore, this work opens an avenue for developing novel metal cluster‐based photocatalysts via a thermally induced covalent crosslinking strategy for promising solar energy conversion.

## Conflict of Interest

The authors declare no conflict of interest.

## Supporting information



Supporting Information

## Data Availability

The data that support the findings of this study are available in the supplementary material of this article.
