# Supplementary material for: New Strategy for Cluster‐Based Covalent Organic Framework: Thermally Induced Covalent Crosslinking of Highly Stable Copper Clusters
Source: Adv Sci (Weinh). 2025 Jul 30;12(35):e07510. doi: 10.1002/advs.202507510 (PMC12463109; doi:10.1002/advs.202507510)
Supplement: Supplementary file 1 — Supporting Information [file ADVS-12-e07510-s001.docx]

**Supporting Information**

**New Strategy for Cluster-Based Covalent Organic Framework: Thermally Induced Covalent Crosslinking of Highly Stable Copper Clusters**

Jian-Peng Dong^a,b#^, Yue Xu^a#^, Ling Yao^a^, Le Wang^a^, Gang Li^a^, Rui Wang^a*^ and Shuang-Quan Zang^a*^

^a^Henan Key Laboratory of Crystalline Molecular Functional Materials, Green Catalysis Center, and College of Chemistry, Zhengzhou University, Zhengzhou, 450001, P. R. China.

^b^College of Chemistry and Pharmaceutical Engineering, Nanyang Normal University, Nanyang, 473061, P. R. China.

^#^These authors contributed equally to this work.

^*^Corresponding author: Rui Wang; Shuang-Quan Zang.

E-mail: wangruijy@zzu.edu.cn; zangsqzg@zzu.edu.cn.

**1. Experimental methods**

**1.1. Materials.**

All chemicals and solvents obtained from suppliers were used without additional purification. 4-(4-Pyridinyl)thiazole-2-thiol (98%, SN), 4-Phenylthiazole-2-thiol (98%, SC) and Cu(CH_3_CN)_4_PF_6_ (97%) were purchased from Energy Chemical Co., Ltd. Nafion 117 solution (5 wt.%) was purchased from Sigma-Aldrich. All other reagents and solvents used were of commercial reagent grade without any additional purification.

**1.2. Instrumentation.**

Powder X-ray diffraction (PXRD) data were collected under the atmosphere using X’Pert PRO diffractometer (Cu–Kα, λ = 1.54178 Å). Thermo-gravimetric analysis/differential scanning calorimetry (TGA/DSC) was performed by a Mettler-Toledo TGA/DSC 3+ under N_2_ atmosphere with a heating rate of 10 °C/min. The X–ray photoelectron spectroscopy (XPS) measurements were tested using ESCALAB 250Xi system (Thermo Electron). The C peak at 284.8 eV was used as a reference to correct for charging effects. The Fourier transform infrared (FT-IR) spectra were recorded on Bruker ALPHA spectrometer. The SPV spectra were conducted on a surface photovoltage spectrometer system between 300-600 nm with a 500 W Xe lamp. The ultraviolet-visible (UV-Vis) diffuse reflectance spectroscopy were collected on UH4150 spectrophotometer using BaSO_4_ as the reference. Transmission electron microscopy (TEM) images were obtained in FEI Talos F200S. High-resolution transmission electron microscopy (HRTEM) images were obtained in JEM–2100 equipment. CO_2_ adsorption isotherms (298 K) were measured by using automatic volumetric adsorption equipment (Belsorp Max) after a degassed process at 120 °C for 12 h. Photoluminescence spectra and luminescence decay was measured on a HORIBA FluoroLog–3 fluorescence spectrometer. Element analyses (EA) were collected on a PerkinElmer 240 elemental analyzer. The inductively coupled plasma mass spectrometry (ICP-MS) analysis was recorded on Optimass 9500. ^1^H NMR spectra were recorded on Bruker AVIII HD 600 instruments at 600 MHz. The solid-state ^13^C cross-polarization magic angle spinning nuclear magnetic resonance (^13^C CP/MAS NMR) spectra were collected over 64 transients with 10 s recycling delay by spinning the sample at 12 kHz measured on a 400 MHz Bruker Avance DSX NMR spectrometer. Electrochemical test was carried out on electrochemical working station CHI 660E (Shanghai Chenhua).

**1.3. Preparation of Cu_4_SN.**

Cu(CH_3_CN)_4_PF_6_ (37.2 mg, 0.1 mmol) was dissolved in 10 mL acetonitrile, Then a methanol solution (10 mL) of SN (19.4 mg, 0.1 mmol) was added. After vigorous stirring at room temperature for 5 min, 50 μL Et_3_N was added, **Cu_4_SN** powder crystals (C_32_H_20_Cu_4_N_8_S_8_) were obtained after stirred for another 30 min (a single crystal suitable for SCXRD measurements can be obtained without the addition of Et_3_N, the resulting solution was then evaporated at room temperature for about three days to afford block crystals). The white powder crystals were isolated and washed with methanol then dried in air at 60 ˚C (yield 99%, based on Cu(CH_3_CN)_4_PF_6_). Elemental analysis for **Cu_4_SN** (C_32_H_20_Cu_4_N_8_S_8_): calcd. C, 37.41%; H, 1.96%; N, 10.91%; S, 24.97%; found: C, 37.33%; H, 1.99%; N, 11.05%; S, 24.82%.

**1.4. Preparation of Cu_4_SC.**

Cu(CH_3_CN)_4_PF_6_ (37.2 mg, 0.1 mmol) was dissolved in 10 mL acetonitrile, Then a methanol solution (10 mL) of SC (19.3 mg, 0.1 mmol) was added. **Cu_4_SC** powder crystals (C_36_H_24_Cu_4_N_4_S_8_) were obtained by filtration after vigorous stirring at room temperature for 30 min. The powder crystals were isolated and washed with methanol then dried in air at 60 ˚C (yield 92%, based on Cu(CH_3_CN)_4_PF_6_). Elemental analysis for **Cu_4_SC** (C_36_H_24_Cu_4_N_4_S_8_): calcd. C, 42.25%; H, 2.36%; N, 5.48%; S, 25.07%; found: C, 42.19%; H, 2.40%; N, 5.37%; S, 25.15%.

**1.5. Preparation of *CC*-Cu_4_SN.**

For ***CC–*Cu_4_SN**, the as-prepared precursor **Cu_4_SN** (20 mg) was heated under a N_2_ (100 mL·min^−1^) atmosphere to 240 °C at a ramping rate of 10 °C·min^−1^ and kept at that temperature for 90 min followed by cooling to room temperature naturally. The sample is labeled ***CC–*Cu_4_SN**.

**1.6. Thermogravimetry Differential Scanning Calorimetry−Mass Spectrometry (TG−DSC−MS).**

TG−DSC−MS analysis was performed using a STA 449 F3 Jupiter (Netzsch Instrument) coupled with a quadrupole mass spectrometer QMS 403 D (Aeolos) to elucidate the decomposition processes of **Cu_4_SN** in N_2_. The measurements were conducted from RT-800 °C with 10 K·min^-1^ gradient, after which the samples were cooled down to room temperature. The weight of initial samples was about 1.57 mg, and the pyrolysis process was conducted under a N_2_ gas flow of 250 mL·min^-1^.

**1.7. Single-Crystal X-ray Diffraction Analysis (SCXRD).**

SCXRD measurement was performed on a Rigaku XtaLAB Pro diffractometer with Cu-K*α* radiation (λ= 1.54184 Å) at 200 K. Data collection and reduction were performed using the program CrysAlisPro.^[1]^ The structures were solved with intrinsic phasing methods (*SHELXT-2015*),^[2]^ and refined by full-matrix least squares on *F*^2^ using *OLEX*_2_,^[3]^ which utilizes the *SHELXL-2015* module. Imposed restraints in least-square refinement of each structure were commented in the corresponding CIF files. Thus, only a general description of the structural refinement strategy is presented here. All non-hydrogen atoms were refined anisotropically, and the hydrogen atoms were included on idealized positions. Pertinent crystallographic data collection and refinement parameters are collated in Table S1. The crystal structures are visualized by DIAMOND 3.2.^[4]^

**1.8. X-ray Crystallography of *CC–*Cu_4_SN.**

Because no single crystal specimen of diffraction quality was able to be produced for ***CC–*Cu_4_SN**, the crystal structure was determined by Rietveld refinement of its PXRD data. The PXRD patterns were collected (0.01 ˚/step, 0.2 seconds/step) on a X’Pert PRO diffractometer (Cu–Kα, λ = 1.54178 Å) at room temperature. All analysis of powder diffraction data including Rietveld refinement was carried out using the TOPAS Academic 7.0 software in the 2 *θ* range of 3–50˚ on unit-cell parameters,^[5]^ yielding the following parameters: *R*_p_ = 3.84%, *R*_wp_ = 5.43% for ***CC–*Cu_4_SN**. Crystallographic data were summarized in Table 5.

**1.9. XAFS measurement and data analysis.**

The XAFS spectra were obtained at the 1W1B station in Beijing Synchrotron Radiation Facility (BSRF), China. XAFS measurements at the Cu K-edge were recorded in a fluorescence mode using a Lytle detector. Cu foil, CuPc, Cu_2_S and CuS were used as references. The sample were collected several times to obtain high-quality data. Data reduction, data analysis, and EXAFS fitting were applied through Athena and Artemis software.^[6]^ The energy calibration of the sample was conducted through a standard Cu foil, which as a reference was simultaneously measured. For EXAFS modeling, the global amplitude EXAFS (*CN*, *R*, σ^2^ and Δ*E*_0_) were obtained by nonlinear fitting, with least-squares refinement, of the EXAFS equation to the Fourier-transformed data in R-space, using Artemis software, EXAFS of the Cu foil was fitted and the obtained amplitude reduction factor *S_0_^2^* value was set in the EXAFS analysis to determine the coordination numbers (CNs) in the scattering path in samples. Wavelet transformation (WT) was also employed using the software package developed by Funke and Chukalina using Morlet wavelet with κ = 10, σ = 1.^[7]^

In situ X-ray absorption fine structure (XAFS) analyses of the Cu K-edge were conducted utilizing a commercial Laboratory-Based XAFS spectrometer (RapidXAFS 2M, Anhui Absorption Spectroscopy Analysis Instrument Co., Ltd.). X-rays were generated via a Mo target X-ray source operated at 20 kV and 20 mA. A Si (553) spherically bent crystal analyzer (SBCA) with a radius of curvature of 500 mm served as the monochromator. The XAFS data were acquired in transmission mode. For the Photocatalytic in situ XAFS tests, the samples were pressed into pellets using a pellet press and placed into a custom-made cell. The X-ray absorption spectra were measured under three different conditions: the initial state of the sample under an Ar atmosphere, under a saturated CO_2_ atmosphere containing water vapor, and under a saturated CO_2_ atmosphere with illumination. The oxidation state changes of the samples were analyzed by examining the Cu K-edge X-ray Absorption Near Edge Structure (XANES) spectra. XAFS data analysis was based on standard data reduction using the ATHENA (v0.9.26) software packages.

**1.10. Electrochemical measurements.**

All electrochemical measurements (photocurrent, and electrochemical impedance spectroscopy) were carried out at ambient environment using the electrochemical workstation (CHI 660e) in a standard three-electrode system. The working electrodes for photocurrent and electrochemical impedance spectroscopy were prepared as follows: 1 mg sample powder was dispersed into 5 μL 5 wt% Nafion and 0.5 mL ethanol mixed solution, and sonicated for 30 minutes. The working electrode was prepared by dropping the suspension (5 μL) onto the surface of the glassy carbon electrode and dried in ambient condition. Electrochemical impedance spectroscopy (EIS) was performed in the frequency range from 0.1 Hz to 10^5^ Hz. A 300W Xenon lamp was used as the incident light in the photocurrent measurements.

**1.11. Photocatalytic Experiments.**

The photoreduction of CO_2_ with H_2_O was done in a 50 mL quartz reactor. In a typical test, two milligrams of photocatalyst were first dispersed in 10 mL aqueous solution through ultrasonic for 30 min to obtain the uniform dispersion. After bubbling with high-purity CO_2_ for 30 min to remove all the air, the reactor was sealed and treated with light irradiation. During the photoreduction process, the reactor was irradiated with a 300 W Xe lamp under vigorous stirring and the light intensity is tested as 150 mW cm^–2^. The reaction temperature was maintained at 25 ˚C using the recycling cooling water. All product gases were analyzed via an Agilent 8890 gas chromatography equipped with a flame ionization detector (FID) and thermal conductivity detector (TCD). The liquid products were quantified by nuclear magnetic resonance (NMR) (Bruker AVIII HD 600 MHz) spectroscopy using maleic acid as the internal standard (ISTD). In isotope experiments, ^13^CO_2_ was used to replace ^12^CO_2_, and the gas products were analyzed by gas chromatography-mass spectrometry (GCMS-QP2010, Shimadzu).

**1.12. Photocatalytic Experiments Apparent quantum efficiency (AQE) calculations.**

Different band-pass filters (centered at 365 nm, 380 nm, 420 nm, 435 nm, 450 nm, 475 nm, 500 nm, 520 nm) were equipped when conducting reactions under photons of different wavelengths and collecting AQE results. Depending on the amount of CO produced by the photocatalytic reaction in an average of two hours, and the AQE was calculated as follow:

$$AQE= \frac{N_{e}}{N_{p}}\times100\%= \frac{2\times M \times N_{A} \times h \times c}{S \times P \times t \times\lambda}$$

where N_e_ is the amount of reaction electrons, N_p_ is the incident photons, M is the amount of CO molecules, N_A_ is Avogadro constant, h is the Planck constant, c is the speed of light, S is the irradiation area, P is the intensity of the irradiation, t is the photoreaction time and λ is the wavelength of the monochromatic light.

**1.13. In situ diffuse reflectance infrared Fourier transform spectroscopy (DRIFTS) measurements.**

In situ DRIFTS were obtained by using a Bruker INVENIO S FT-IR spectrophotometer, equipped with an MCT detector cooled by liquid nitrogen and a commercial reaction chamber from Harrick Scientific. The sample was degassed at 373 K in Ar atmosphere for 30 min to remove water and impurities on the material. Temperature was lowered to room temperature and background spectra were recorded, CO_2_+H_2_O mixture was then injected into the cell and spectra were recorded with background being subtracted. Each spectrum was recorded by averaging 64 scans at a 4 cm^-1^ spectral resolution.

**1.14. Gibbs free energy computational methods.**

The Vienna Ab Initio Package (VASP) was employed to perform all the DFT calculations within the generalized gradient approximation (GGA) using the PBE formulation.^[8]^ The projected augmented wave (PAW) potentials were applied to describe the ionic cores and take valence electrons into account using a plane wave basis set with a kinetic energy cutoff of 450 eV.^[9]^ Partial occupancies of the Kohn–Sham orbitals were allowed using the Gaussian smearing method and a width of 0.05 eV. The electronic energy was considered self-consistent when the energy change was smaller than 10^−5^ eV. A geometry optimization was considered convergent when the force change was smaller than 0.05 eV/Å. Grimme’s DFT-D3 methodology was used to describe the dispersion interactions.^[10]^ The Brillouin zone integral utilized the surfaces structures of 1×1×1 monkhorst pack K-point sampling. Finally, the adsorption energies (Eads) were calculated as E_ads_= E_ad/sub_ -E_ad_ -E_sub_, where E_ad/sub_, E_ad_, and E_sub_ are the total energies of the optimized adsorbate/substrate system, the adsorbate in the structure, and the clean substrate, respectively. The free energy was calculated using the equation:

G=E_ads_+ZPE-TS

where G, Eads, ZPE and TS are the free energy, total energy from DFT calculations, zero point energy and entropic contributions, respectively.

**1.15. Statistical Analysis.**

Mean ± standard deviation (SD) was used to present the CO selectivity, CO and O_2_ evolution rate results. Each set of data was repeated at least three times.

**Table S1.** Crystal data and structure refinements of **Cu_4_SN**.

|  | **Cu_4_SN** |
| --- | --- |
| CCDC number | 2079536 |
| Empirical formula | C_32_H_20_Cu_4_N_8_S_8_ |
| Formula weight | 1027.24 |
| Temperature/K | 200(10) |
| Crystal system | teragonal |
| Space group | *I4_1_/a* |
| *a* / Å | 16.34010(10) |
| *b* / Å | 16.34010(10) |
| *c* / Å | 14.7588(2) |
| *α* / ° | 90 |
| *β* / ° | 90 |
| *γ* / ° | 90 |
| *Volume* / Å^3^ | 3940.58(7) |
| *Z* | 4 |
| *ρ*_calc_ g / cm^3^ | 1.866 |
| *μ* / mm^-1^ | 6.789 |
| F(000) | 2208.0 |
| Crystal size / mm^3^ | 0.3 × 0.1 × 0.1 |
| Radiation | Cu Kα (λ = 1.54184) |
| 2Θ range for data collection / ° | 8.072 to 148.646 |
| Index ranges | -20 ≤ *h* ≤ 17,  -18 ≤ *k* ≤ 17,  -18 ≤ *l* ≤ 9 |
| Reflections collected | 5554 |
| Independent reflections | 1957  [R *_int_* = 0.0198,  R *_sigma_* = 0.0213] |
| Goodness-of-fit on F^2^ | 1.063 |
| Final *R* indexes [I >= 2σ (I)] | R_1_ = 0.0298, *w*R_2_ = 0.0714 |
| Final *R* indexes [all data] | R_1_ = 0.0274, *w*R_2_ = 0.0705 |
| Largest diff. peak/hole / e Å^-3^ | 0.32/-0.35 |

*R*_1_ = ∑׀׀*F*_o_׀−׀*F*_c_׀/∑׀*F*_o_׀ . *wR*_2_ = [∑*w*(*F*_o_^2^ −*F*_c_^2^)^2^/∑*w*(*F*_o_^2^)^2^]^1/2^

**Table S2.** Selected bond distances (Å) for **Cu_4_SN**.

| Cu1-Cu1^1^ | 2.7155(5) | N2-C8 | 1.319(3) |
| --- | --- | --- | --- |
| Cu1-Cu1^2^ | 2.7156(5) | N6-C3 | 1.474(3) |
| Cu1-Cu1^3^ | 2.8810(6) | C6-C7 | 1.357(3) |
| Cu1-S1^1^ | 2.2805(6) | C3-C2 | 1.389(3) |
| Cu1-S1^3^ | 2.2548(6) | C3-C4 | 1.390(4) |
| Cu1-N2 | 1.9968(18) | C2-C1 | 1.388(4) |
| S1-C8 | 1.742(2) | N1-C1 | 1.331(4) |
| S2-C8 | 1.718(2) | N1-C5 | 1.323(4) |
| S2-C7 | 1.707(3) | C4-C5 | 1.379(4) |
| N2-C6 | 1.387(3) |  |  |

1: 5/4-Y,1/4+X,1/4-Z; 2: -1/4+Y,5/4-X,1/4-Z; 3:1-X,3/2-Y,+Z.

**Table S3.** Inductively coupled plasma mass spectrometry (ICP-MS) (Cu) and elemental analysis (EA) (C, H, N, and S) of **Cu_4_SN** and ***CC–*Cu_4_SN**.

| Sample | C [%] | H [%] | N [%] | S [%] | Cu [%] |
| --- | --- | --- | --- | --- | --- |
| **Cu_4_SN** [ C_32_H_20_Cu_4_N_8_S_8_] (Theoretical) | 37.41 | 1.96 | 10.91 | 24.97 | 24.74 |
| **Cu_4_SN** [ C_32_H_20_Cu_4_N_8_S_8_] (Measured) | 37.33 | 1.99 | 11.05 | 24.82 | 24.79 |
| ***CC–*Cu_4_SN** (Theoretical) | 37.45 | 1.87 | 10.92 | 24.99 | 24.77 |
| ***CC–*Cu_4_SN** (Measured) | 37.46 | 1.86 | 10.89 | 25.02 | 24.80 |

**Table S4.** Assignments of peaks observed in TG−DSC−MS spectra of **Cu_4_SN**.

| m/z | Chemical formula | Parent molecule |
| --- | --- | --- |
| 26 | C_2_H_2_ | SN |
| 39 | C_3_H_3_ | SN |
| 58 | C_1_N_1_S_1_/ C_2_S_1_H_2_ | SN |
| 60 | C_3_N_1_S_1_H_2_ | SN |
| 76 | C_5_N_1_H_2_ | SN |
| 77 | C_5_N_1_H_3_ | SN |
| 78 | C_5_N_1_H_4_ | SN |
| 82 | C_3_N_1_S_1_ | SN |
| 91 | C_6_N_1_H_5_ | SN |
| 134 | C_7_N_1_S_1_H_4_ | SN |
| 135 | C_7_N_1_S_1_H_5_ | SN |
| 162 | C_8_N_2_S_1_H_6_ | SN |

**Table S5.** Crystallographic data for ***CC–*Cu_4_SN**.

| Compound | ***CC–*Cu_4_SN** |
| --- | --- |
| Refinement method | Rietveld |
| Space group | *P*_1_c_1_ |
| *a* / Å | 21.35 |
| *b* / Å | 10.88 |
| *c* / Å | 14.25 |
| *α* / ° | 90.00 |
| *β* / ° | 87.84 |
| *γ* / ° | 90.00 |
| *Volume* / Å^3^ | 3307.7 |
| *R*_wp_ (%) | 5.43 |
| *R*_p_ (%) | 3.84 |

**Table S6.** EXAFS fitting parameters (*Ѕ*_0_^2^=0.88 from Cu-foil).

|  | shell | CN*^a^* | R*^b^*(Å) | σ^2^*^c^*(Å^2^) | ΔE_0_*^d^*(eV) | R factor |
| --- | --- | --- | --- | --- | --- | --- |
| Cu-foil | Cu-Cu | 12 | 2.54±0.01 | 0.0087 | 4.6±0.5 | 0.0023 |
| Cu_2_S | Cu-S | 3.1±0.2 | 2.30±0.01 | 0.0095 | 6.6±1.3 | 0.0163 |
| CuS | Cu-S | 4.1±0.1 | 2.25±0.01 | 0.0122 | 1.7±0.9 | 0.0050 |
| CuPc | Cu-N | 4.0±0.3 | 1.94±0.01 | 0.0029 | 7.3±1.8 | 0.0151 |
| Cu_4_SN | Cu-N | 1.0±0.1 | 1.95±0.02 | 0.0057 | 3.8±1.3 | 0.0030 |
|  | Cu-S | 2.0±0.1 | 2.25±0.01 | 0.0069 |  |  |
|  | Cu-Cu | 3.0±0.5 | 2.69±0.02 | 0.0249 |  |  |
| *CC–*Cu_4_SN | Cu-N | 1.0±0.2 | 1.87±0.01 | 0.0103 | -1.8±0.7 | 0.0094 |
|  | Cu-S | 1.5±0.1 | 2.20±0.01 | 0.0044 |  |  |
|  | Cu-Cu | 3.1±0.9 | 2.72±0.01 | 0.0270 |  |  |

*^a^CN*: coordination numbers; *^b^R*: bond distance; *^c^σ*^2^: Debye-Waller factors; *^d^* Δ*E*_0_: the inner potential correction. R factor: goodness of fit. Error bounds that characterize the structural parameters obtained by EXAFS spectroscopy were estimated as CN±20%; R ± 1%; σ^2^ ± 20%.

**Table S7.** Comparison of the performance of ***CC–*Cu_4_SN** with the reported catalysts for CO_2_ reduction with H_2_O.

| Photocatalyst | Selectivity  (CO) | Light intensities (mW/cm^2^) | CO Yield  (μmol g^-1^ h^-1^) | Stability | Ref |
| --- | --- | --- | --- | --- | --- |
| ***CC–*Cu_4_SN** | 99.5% | 150 | 29.98 | 20 cycles,≥120 h | ***This work*** |
| Cu_6_-NH | >99% | 150 | 24.8 | 6 cycles, 36 h | *Angew. Chem. Int. Ed.* **2023**, *62*, e202313648. |
| Cu_1_-TiO_2_/BiVO_4_-4 | ∼100% | N/A | 17.33 | N/A | *J. Am. Chem. Soc.* **2024***, 146,* 9163–9171. |
| Bi-TTCOF-Zn | ∼100% | 400 | 11.56 | 5 cycles, 120 h | *J. Am. Chem. Soc.* **2023**, *145*, 23167–23175. |
| s-BiOBr-mof1 | 93.1% | N/A | 21.96 | 10 cycles, 40 h | *Angew. Chem. Int. Ed.* **2022**, *61*, e202208414. |
| BMO-R | 83.2% | N/A | 8.79 | 3 cycles, 21 h | *Angew. Chem. Int. Ed.* **2022**, *61*, e202212355. |
| ZnSe/CdS DORs | 85% | 100 | 11.3 | 4 cycles, 16 h | *Adv. Mater.* **2022**, *34*,  2106662. |
| Au_SA_/Cd_1-x_S | 62.6% | 600 | 32.2 | 1 cycle, 8 h | *Nat. commun.* **2021**, *12*, 1675. |
| BIO-LOV2 | 99.9% | N/A | 17.33 | 3 cycles, 12 h | *Adv. Mater.* **2020**, *32*,  1908350. |
| BiOCl@Bi_2_O_3_ | ∼100% | N/A | 30 | 3 cycles, 36 h | *Adv. Mater.* **2020**, *32*,  2004311*.* |
| InVO_4_ | N/A | N/A | 18.28 | 1 cycle, 7 h | *J. Am. Chem. Soc.* **2019**, 141, 4209–4213. |
| BNT-OVP | 95.6% | 100 | 20.91 | 4 cycles, 16 h | *Nat. commun.* **2021**, *12*, 4594. |
| Cu-CCN | ∼100% | 200 | 3.086 | 1 cycle, 4.5 h | *ACS Nano* **2020**, *14*, 10552-10561. |
| Mn, C-codoped ZnO | ∼100% | N/A | 0.21 | 4 cycles, 16 h | *Nat. commun.* **2021**, *12*, 4936. |
| 0.7Ni-5OB-CN | 71.8% | N/A | 22.1 | N/A | *Adv. Mater.* **2021**, *33*, 2105482. |

**Table S8.** Inductively coupled plasma mass spectrometry (ICP-MS) (Cu) and elemental analysis (EA) (C, H, N, and S) of ***CC*-Cu_4_SN** before and after 20 continuous cycles (6 h per cycle) of CO_2_RR test.

| Sample | C [%] | H [%] | N [%] | S [%] | Cu [%] |
| --- | --- | --- | --- | --- | --- |
| ***CC*-Cu_4_SN** (Before) | 37.46 | 1.86 | 10.89 | 25.02 | 24.80 |
| ***CC*-Cu_4_SN** (After) | 37.22 | 1.87 | 10.74 | 25.13 | 25.06 |


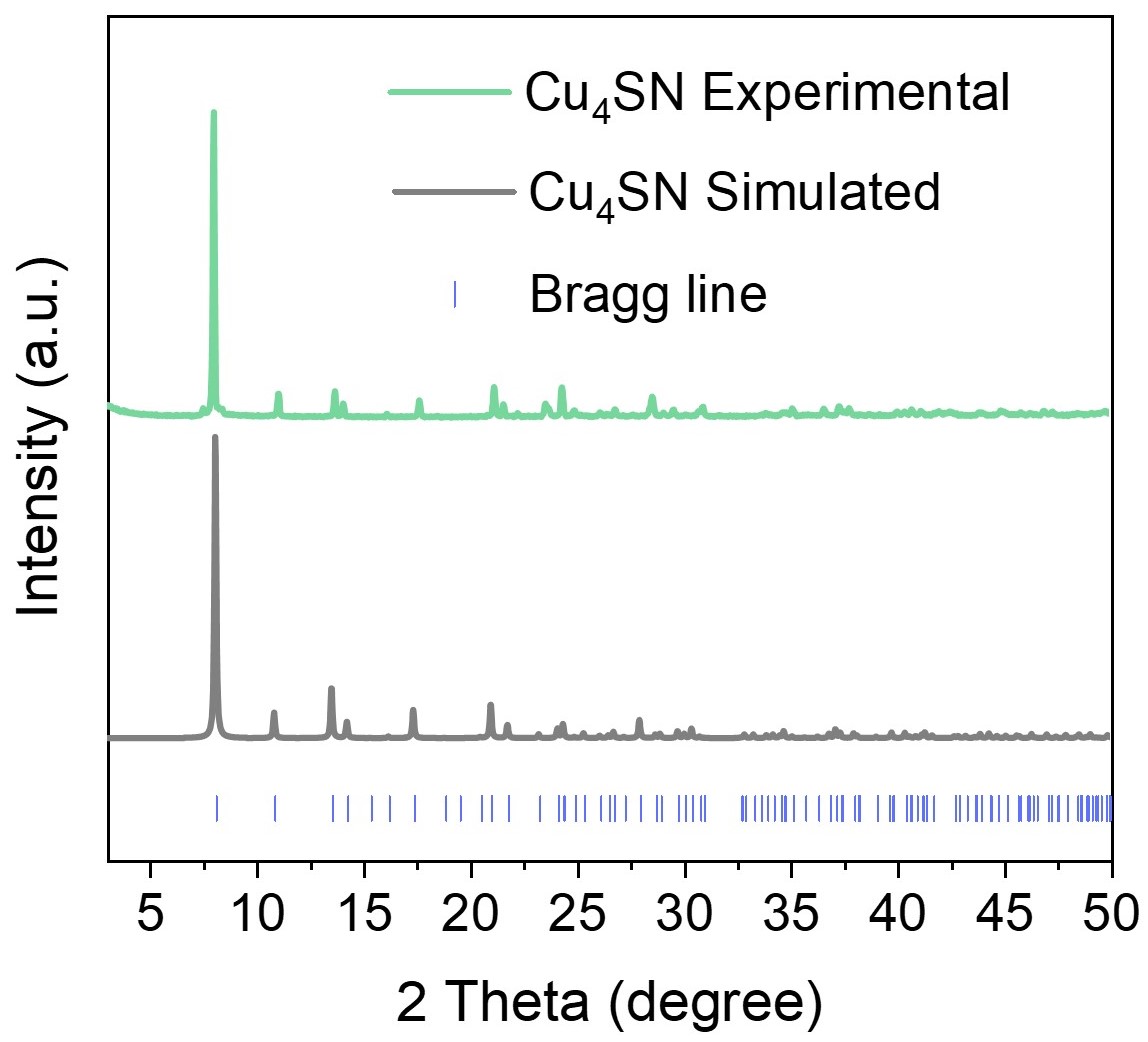


**Figure S1.** PXRD patterns of **Cu_4_SN**.


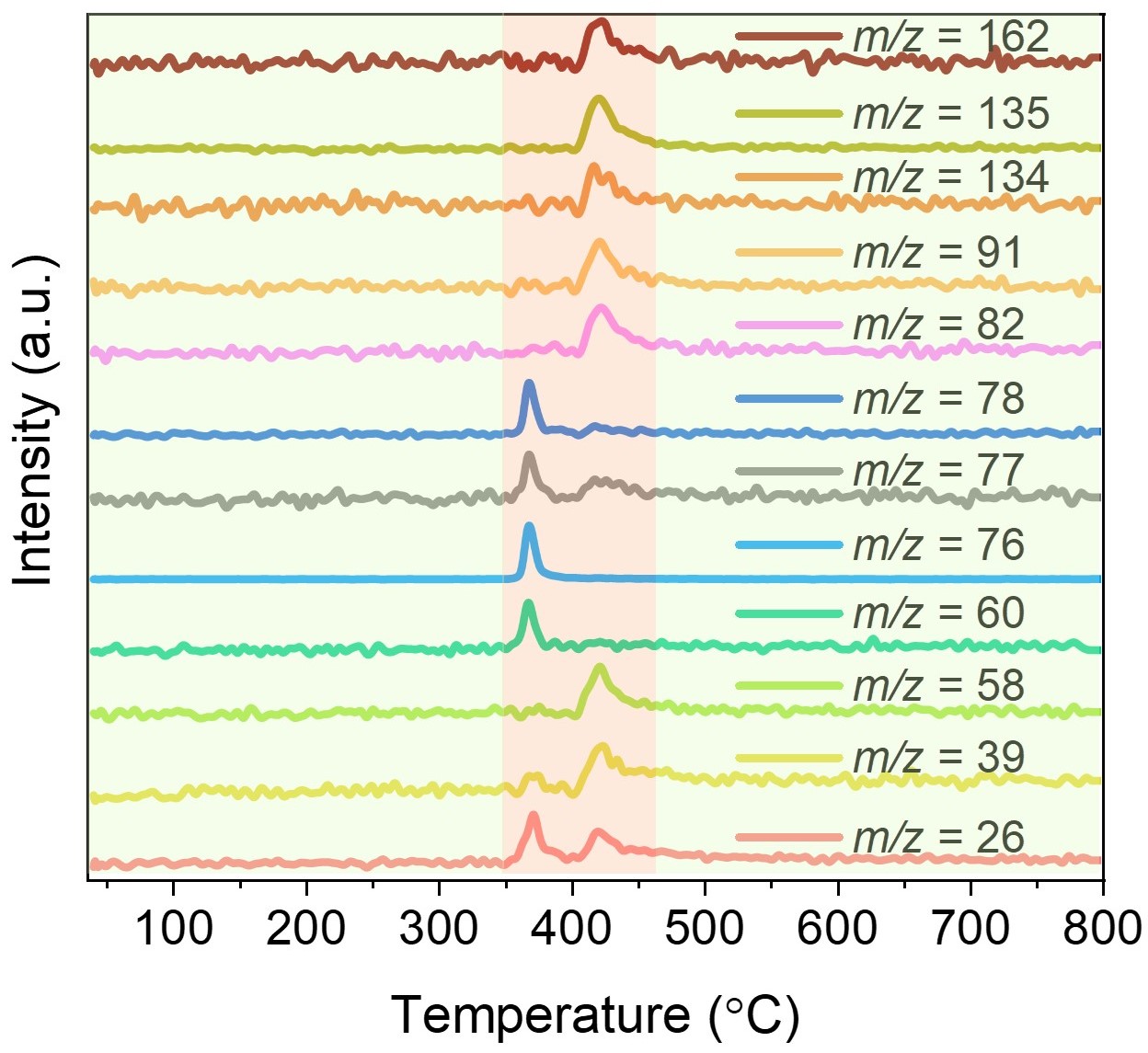


**Figure S2.** The gaseous decomposition products tracked by TG-MS derived from the **Cu_4_SN** pyrolysis process.


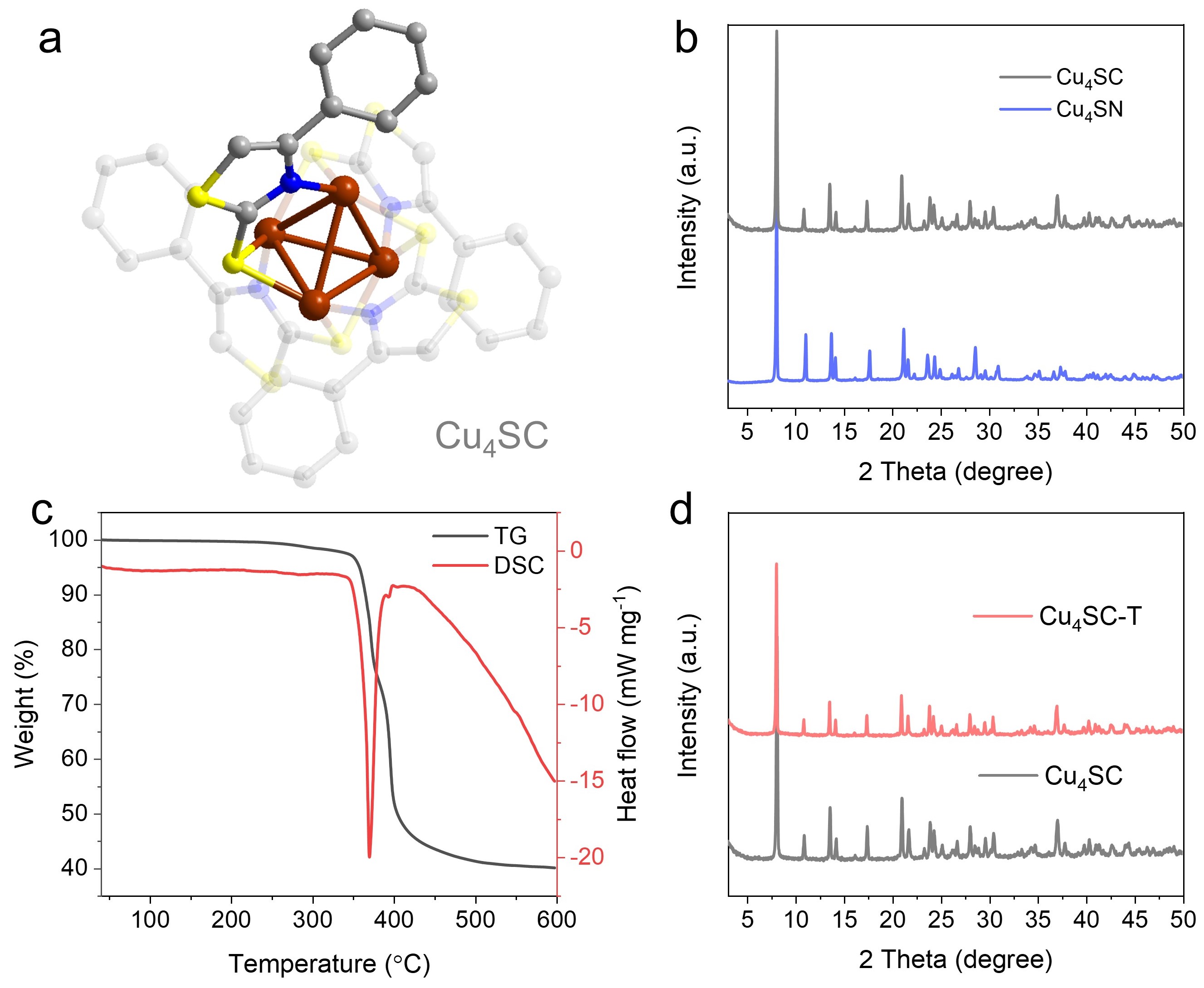


**Figure S3.** (a) The total molecular structure of **Cu_4_SC**. (b) PXRD patterns of **Cu_4_SC**. (c) TG and DSC profile of **Cu_4_SC**. (d) PXRD patterns of **Cu_4_SC** after heat treatment at 240 °C.


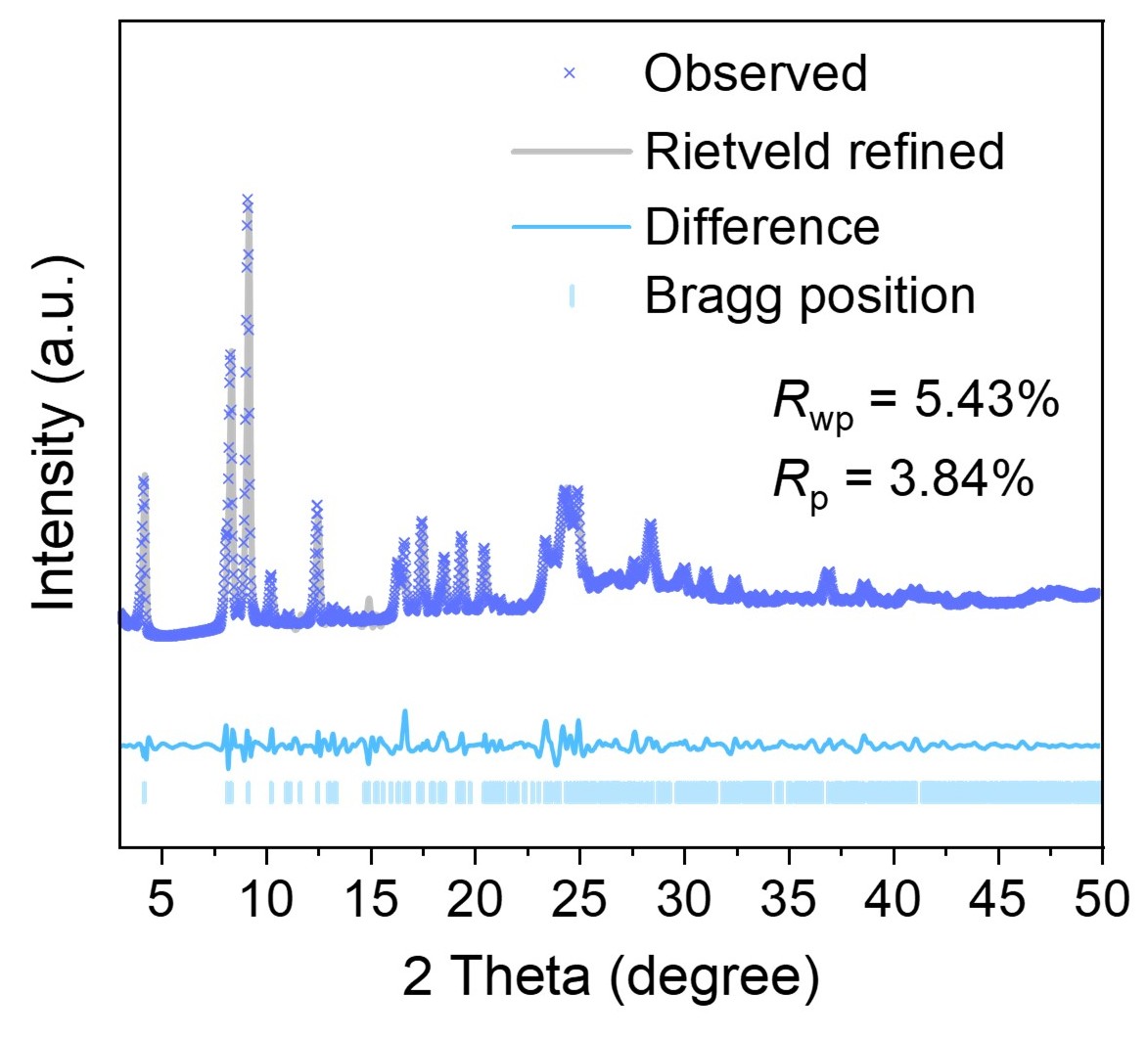


**Figure S4.** Rietveld refinement results of the PXRD pattern of ***CC*–Cu_4_SN**.


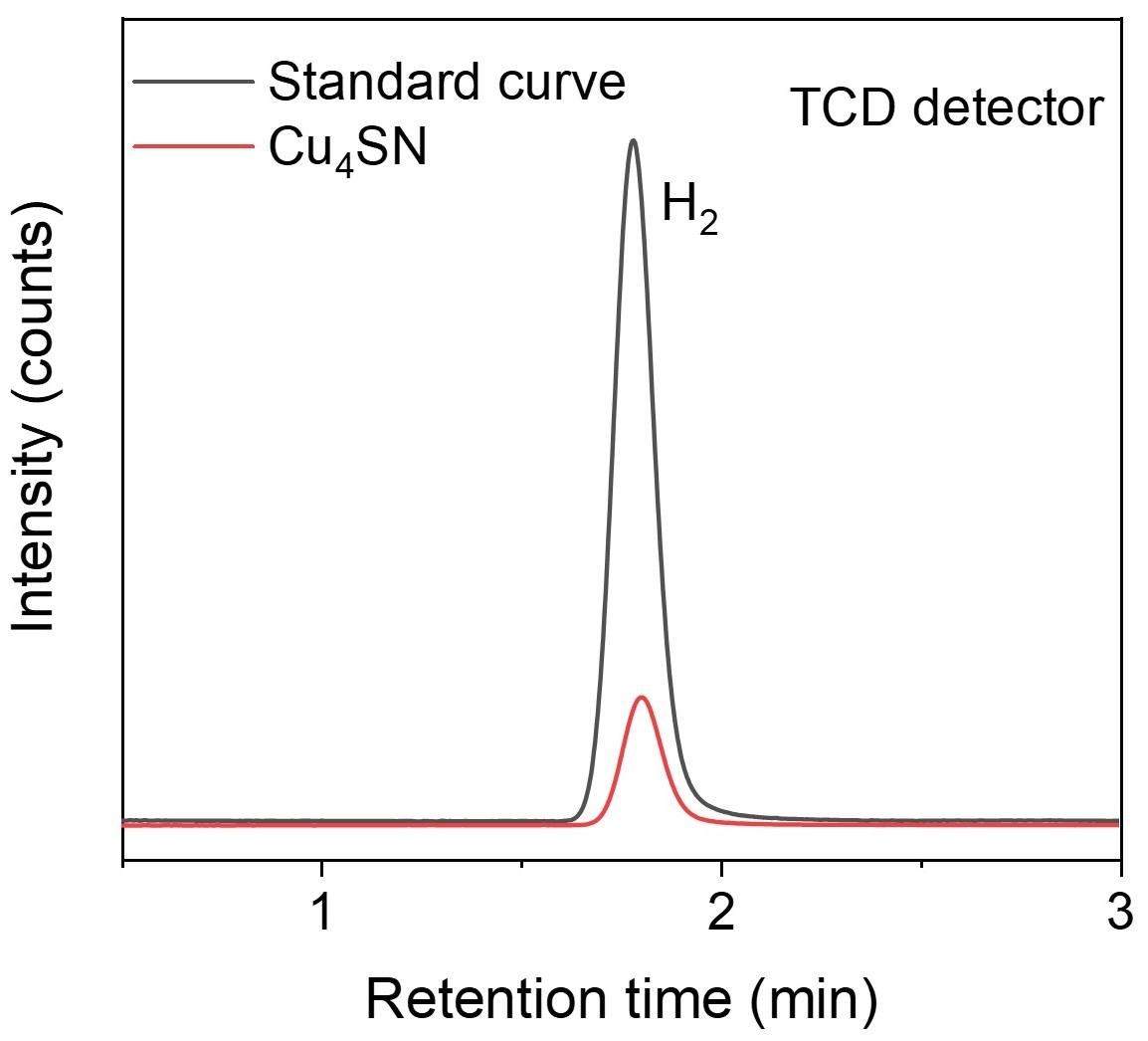


**Figure S5.** Gas chromatograms of products for **Cu_4_SN** treated at 240 °C in nitrogen atmosphere.


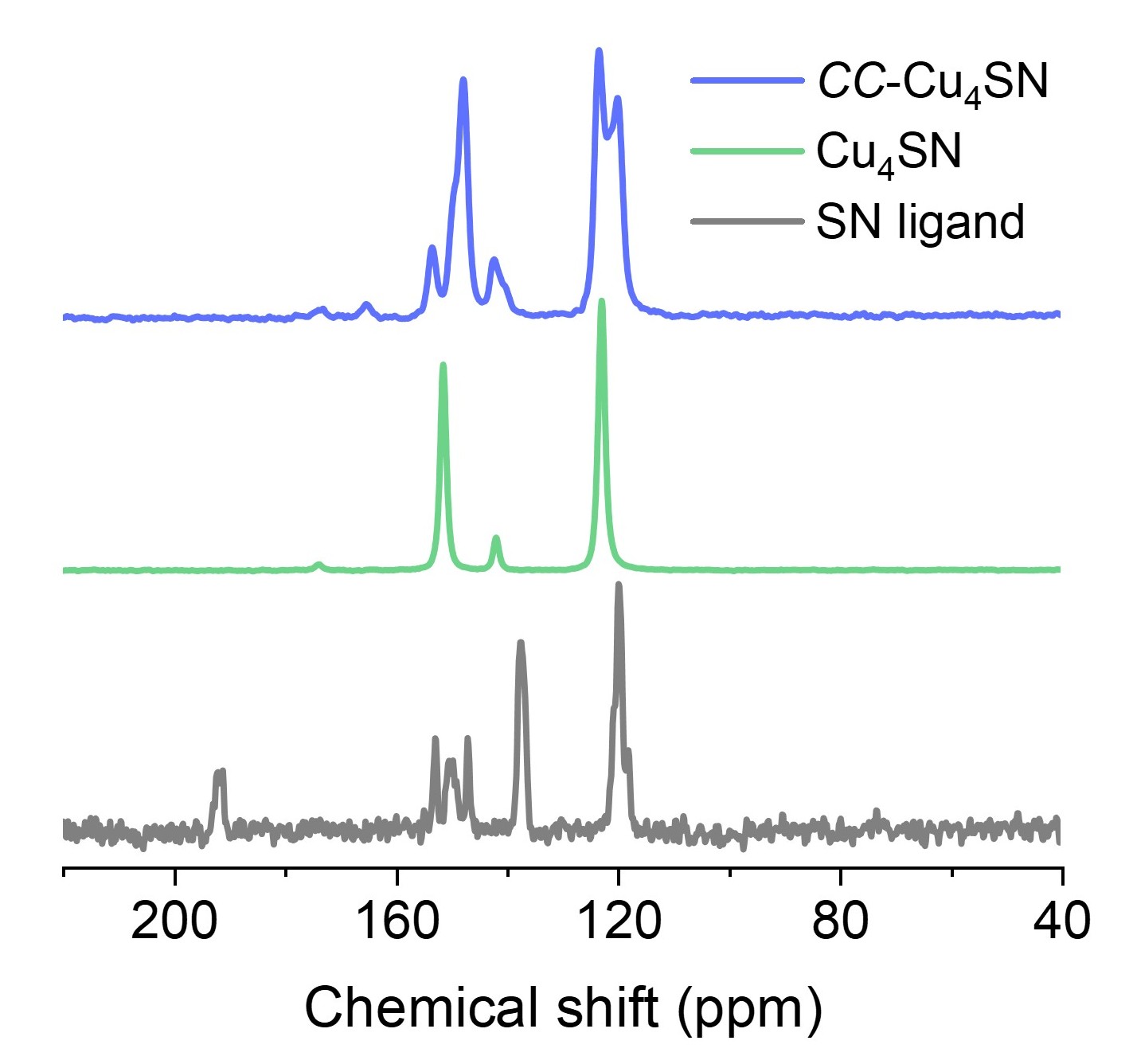


**Figure S6.** ^13^C CP/MAS spectra of SN ligand, **Cu_4_SN** and ***CC*–Cu_4_SN**.


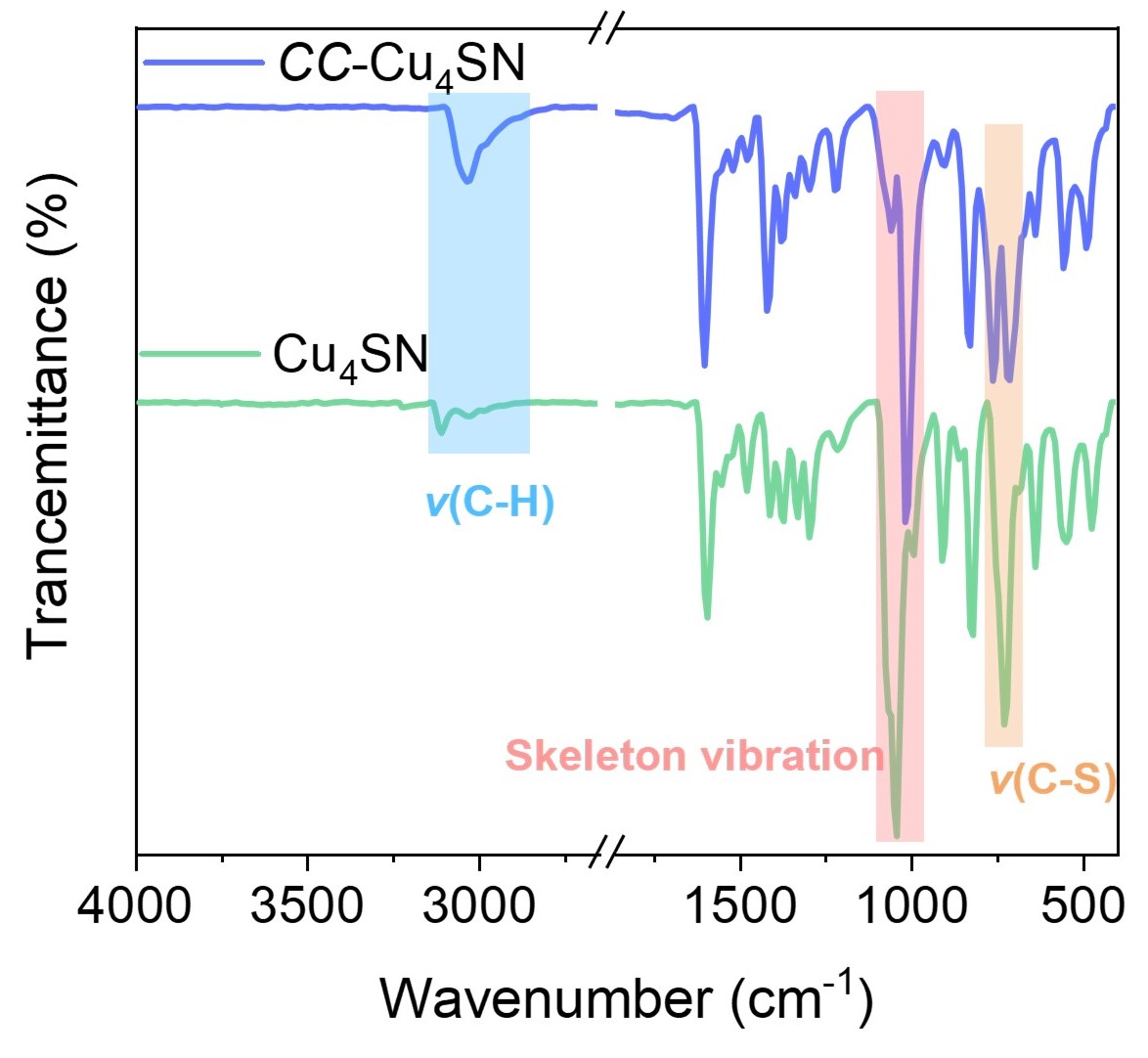


**Figure S7.** FTIR spectra of **Cu_4_SN** and ***CC–*Cu_4_SN**.


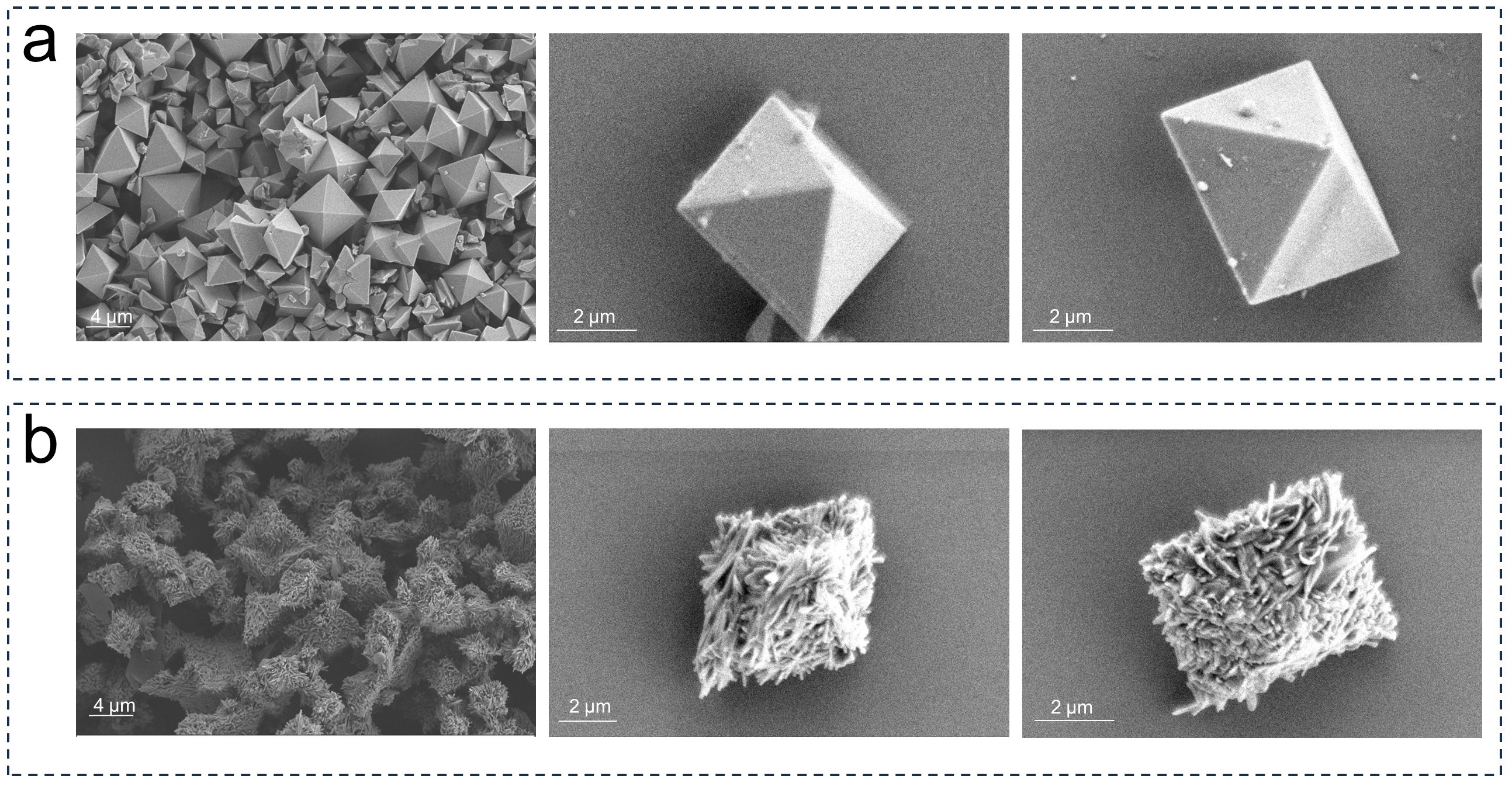


**Figure S8.** SEM images of **Cu_4_SN** (a) and ***CC–*Cu_4_SN** (b).

**
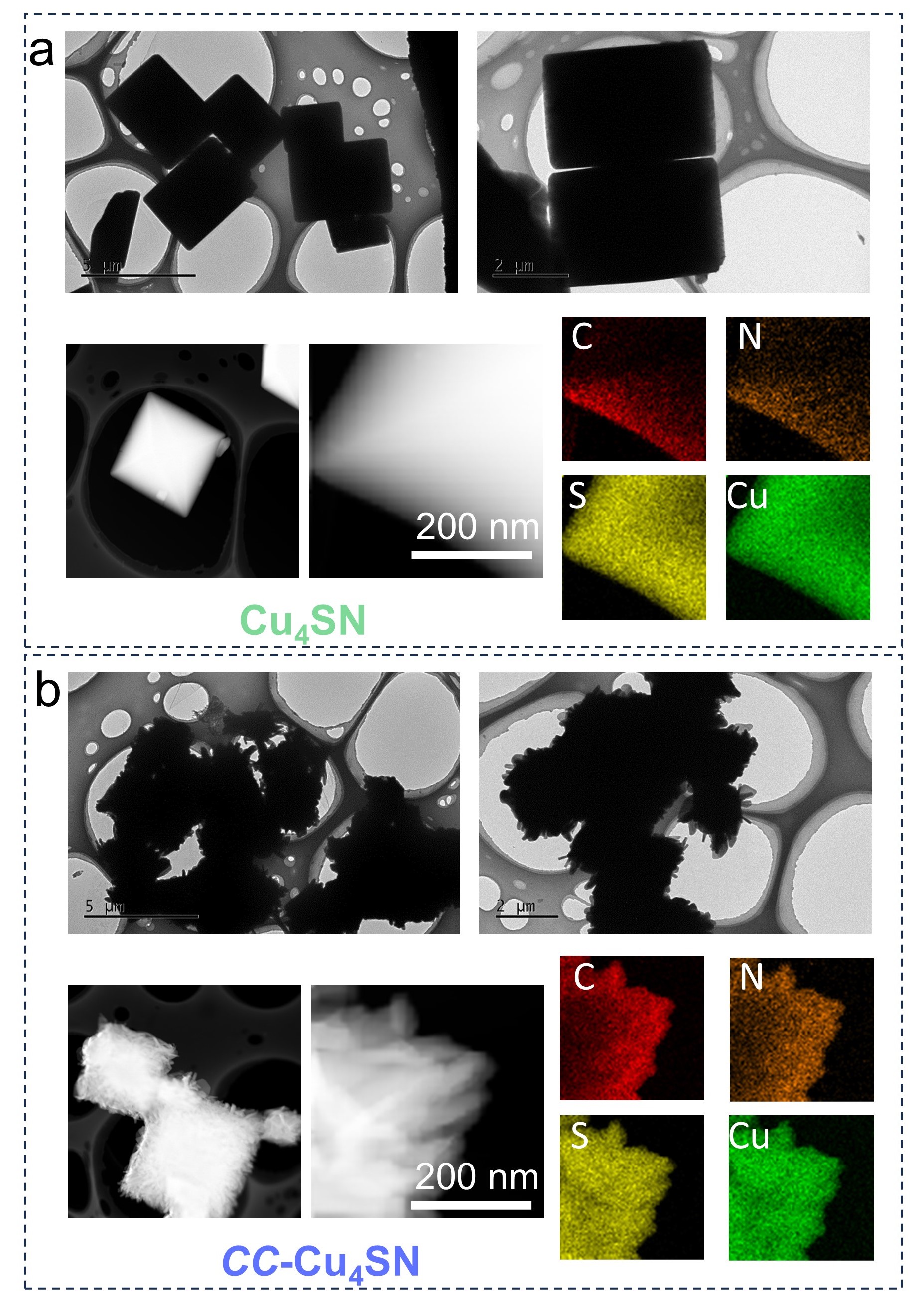
Figure S9.** HRTEM images and annular-dark-field EDX element mapping of **Cu_4_SN** (a) and ***CC–*Cu_4_SN** (b).


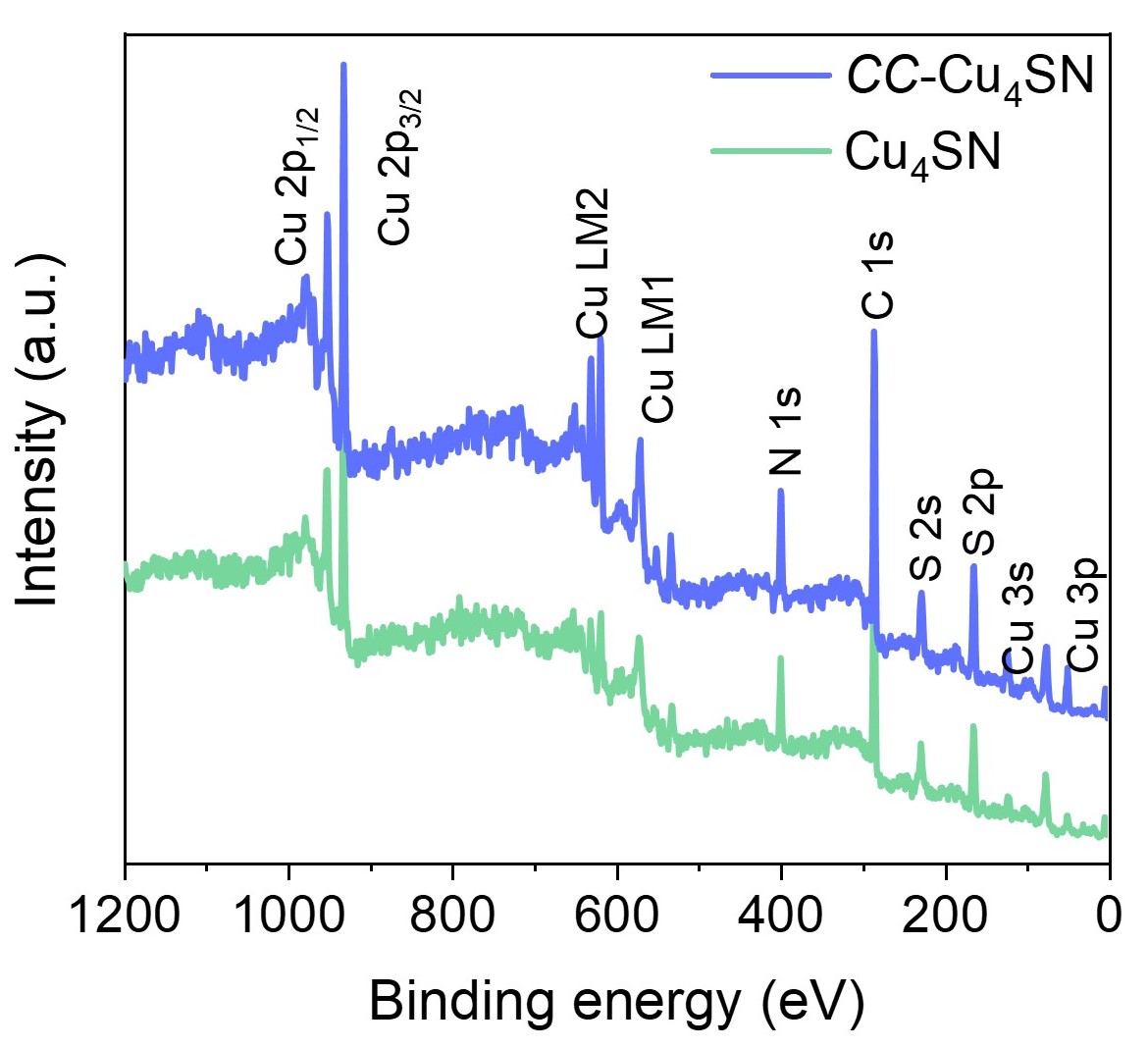


**Figure S10**. XPS survey spectra of **Cu_4_SN** and ***CC–*Cu_4_SN**.


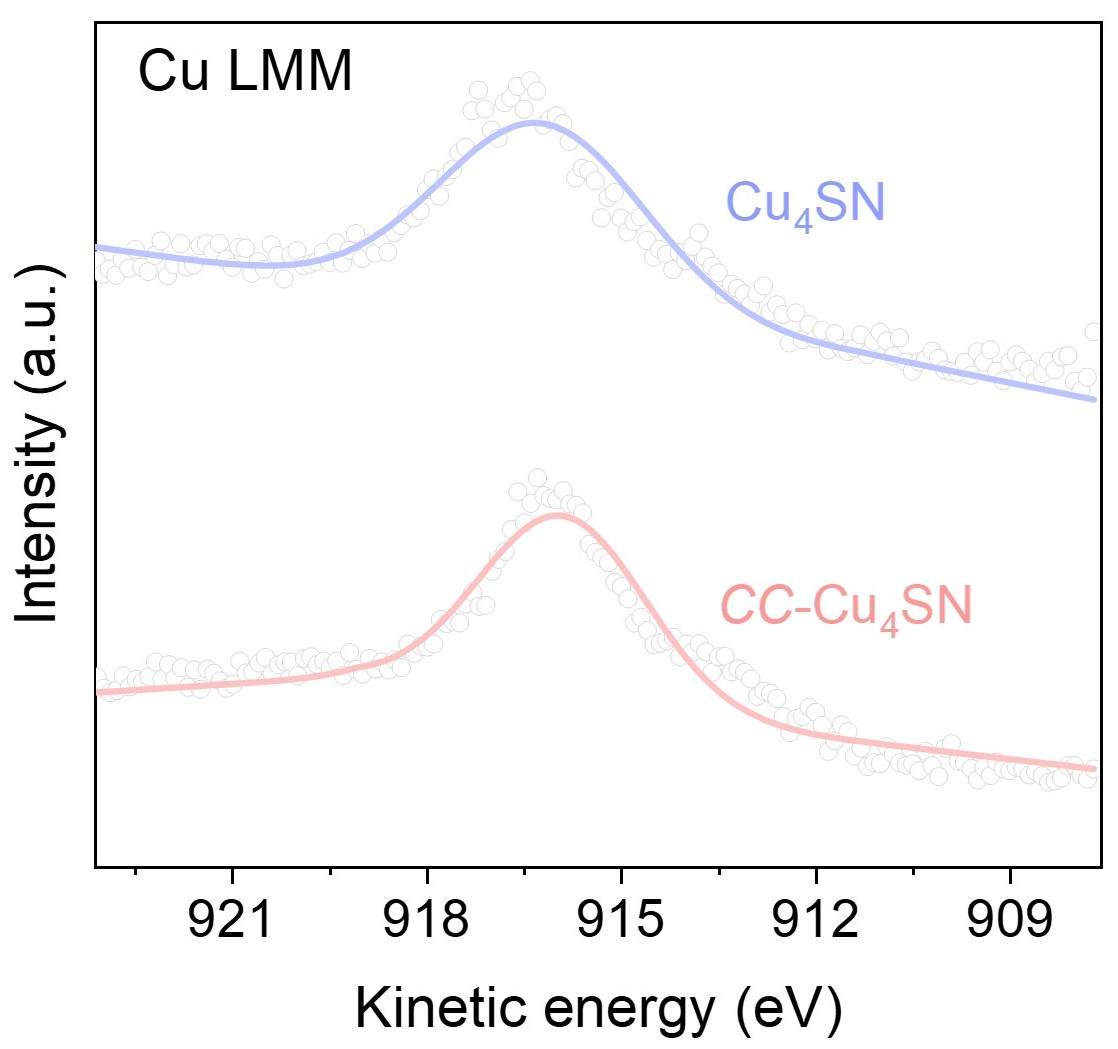


**Figure S11.** Cu LMM Auger spectrum of **Cu_4_SN** and ***CC–*Cu_4_SN**.


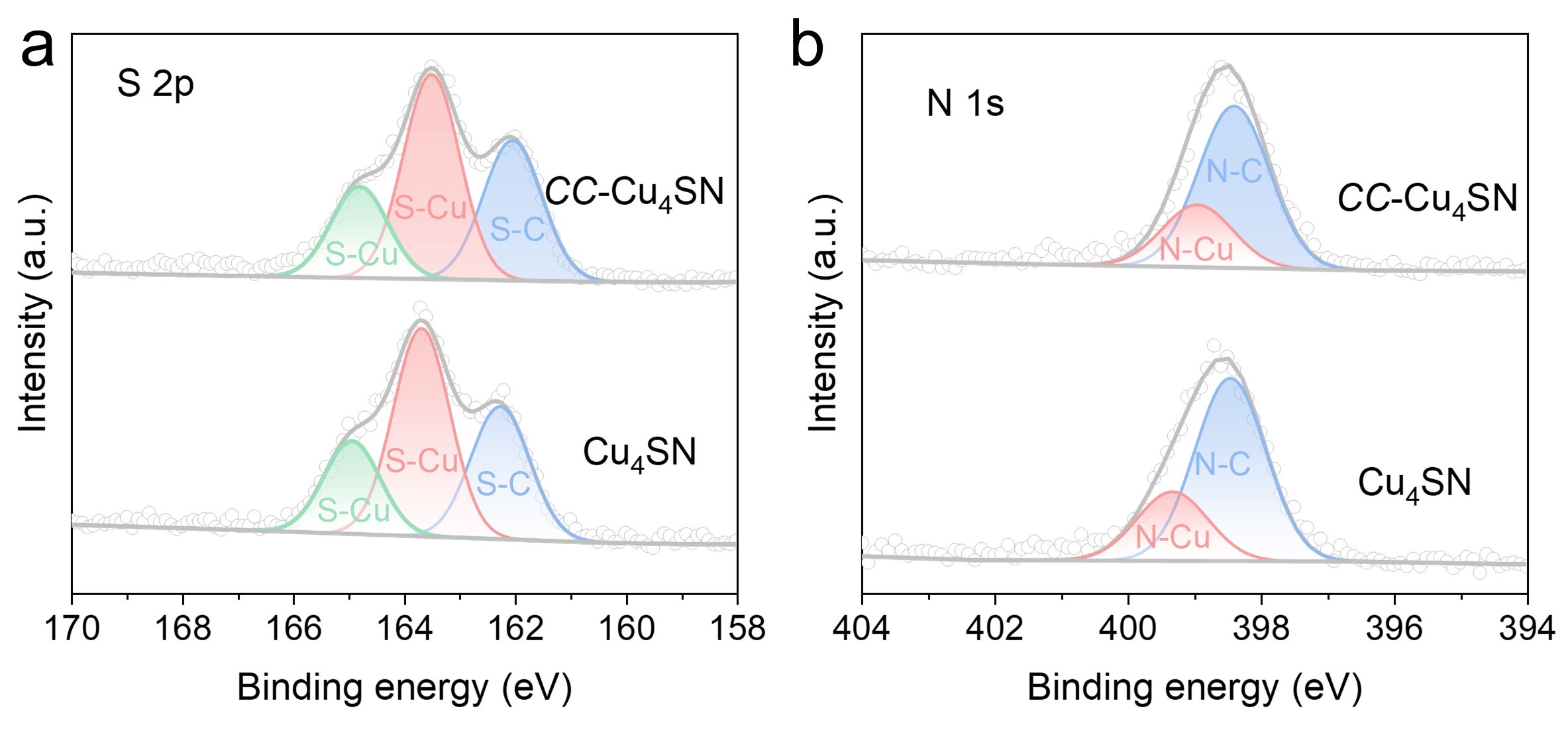


**Figure S12.** XPS S 2p and N 1s spectra of **Cu_4_SN** and ***CC–*Cu_4_SN**.


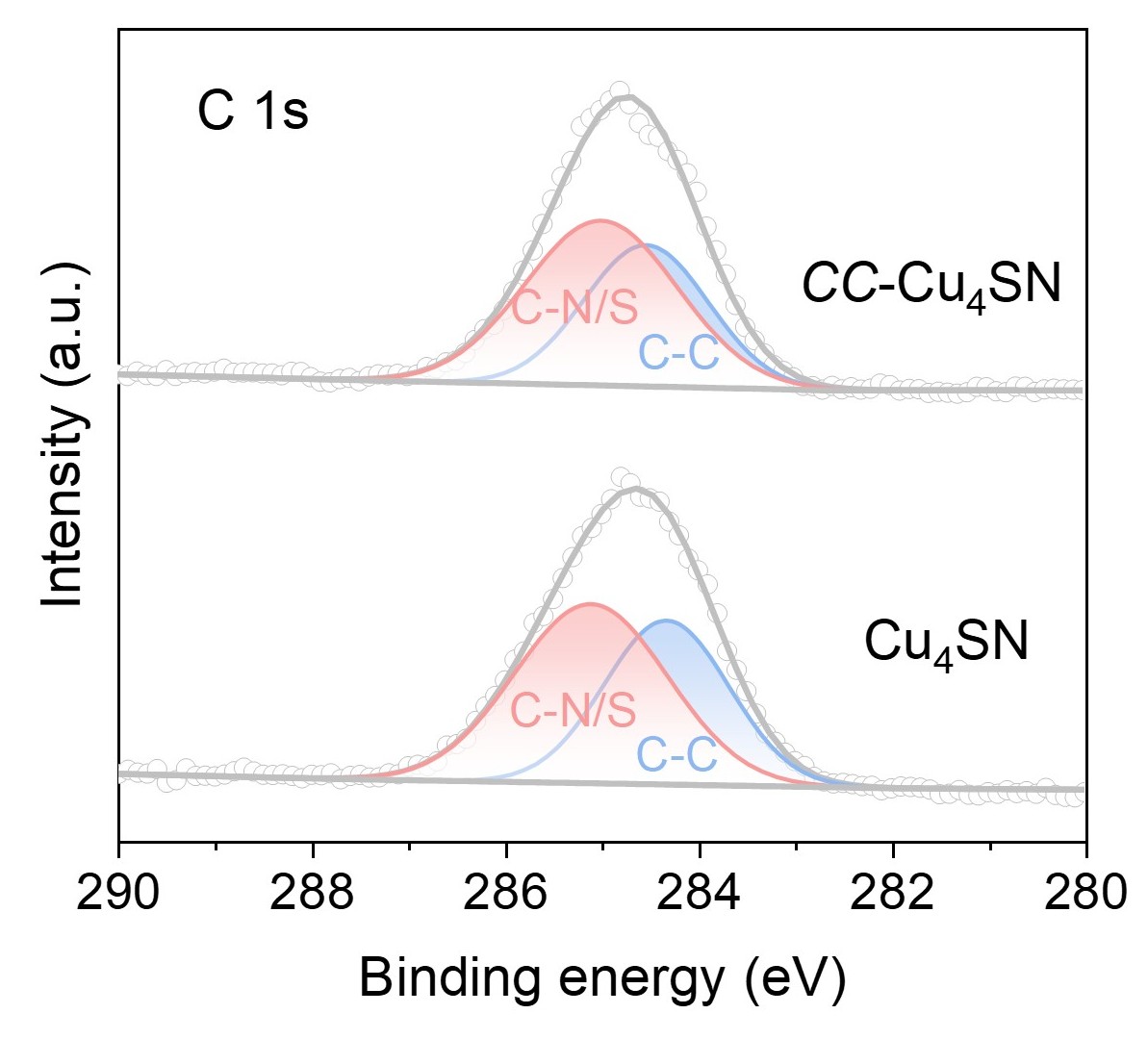


**Figure S13.** High-resolution C 1s XPS spectra of **Cu_4_SN** and ***CC*–Cu_4_SN**.


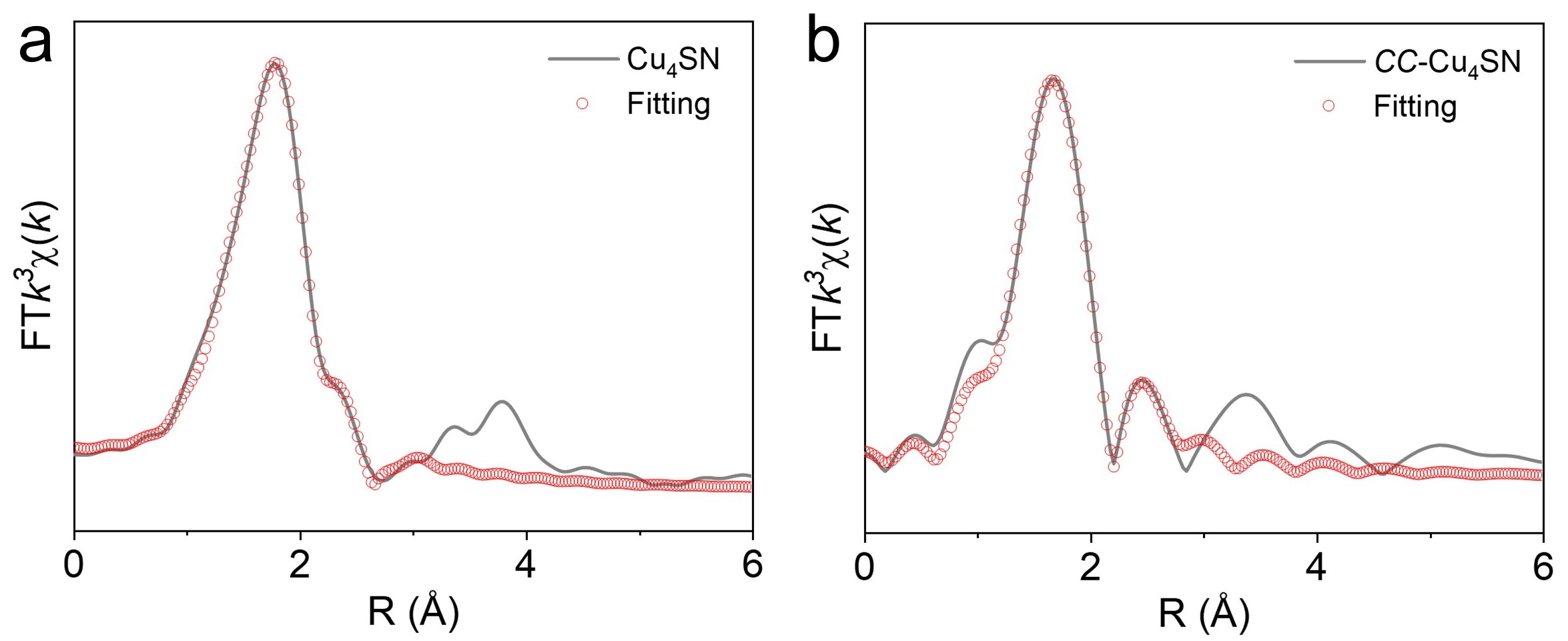


**Figure S14.** Experimental and best-fitted EXAFS spectra in *R* space for **Cu_4_SN** and ***CC–*Cu_4_SN**.


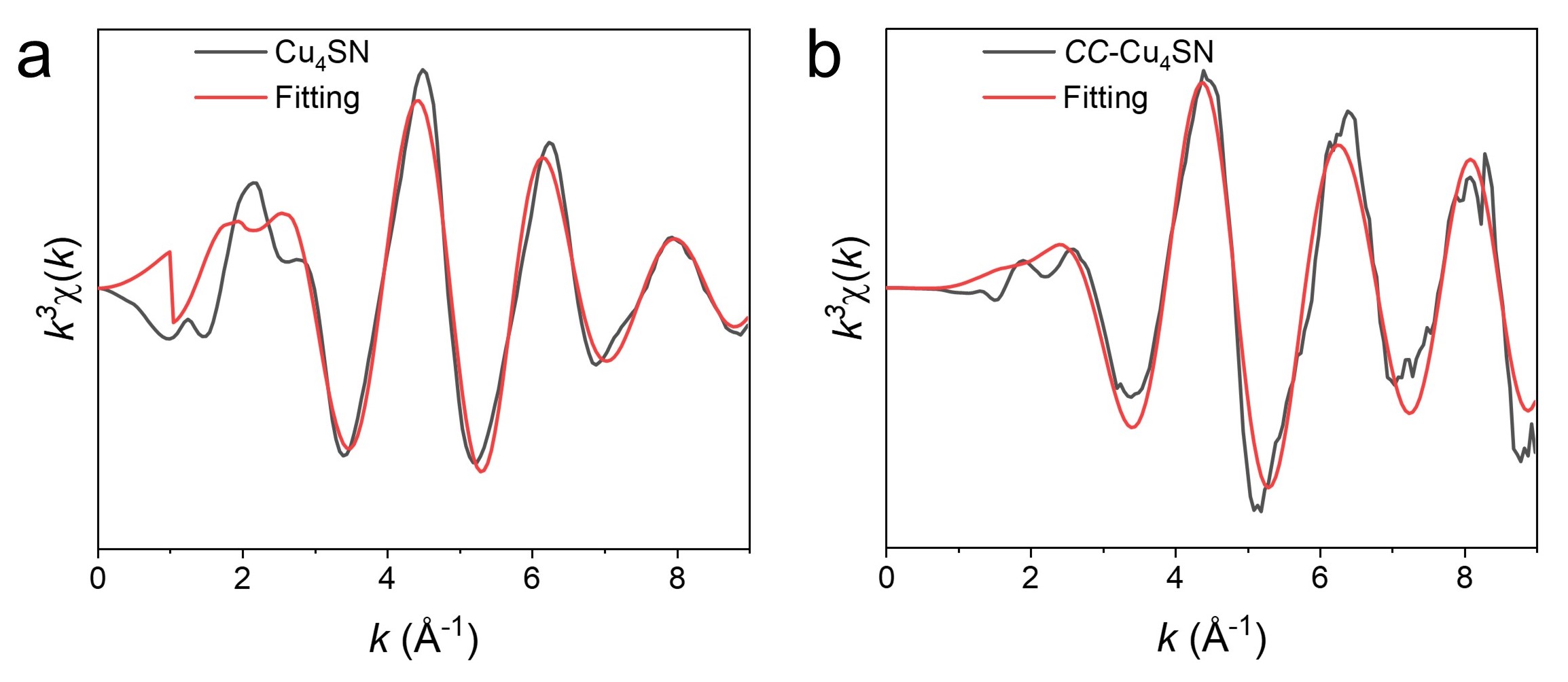


**Figure S15.** Experimental and best-fitted EXAFS spectra in *K* space for **Cu_4_SN** and ***CC–*Cu_4_SN**.


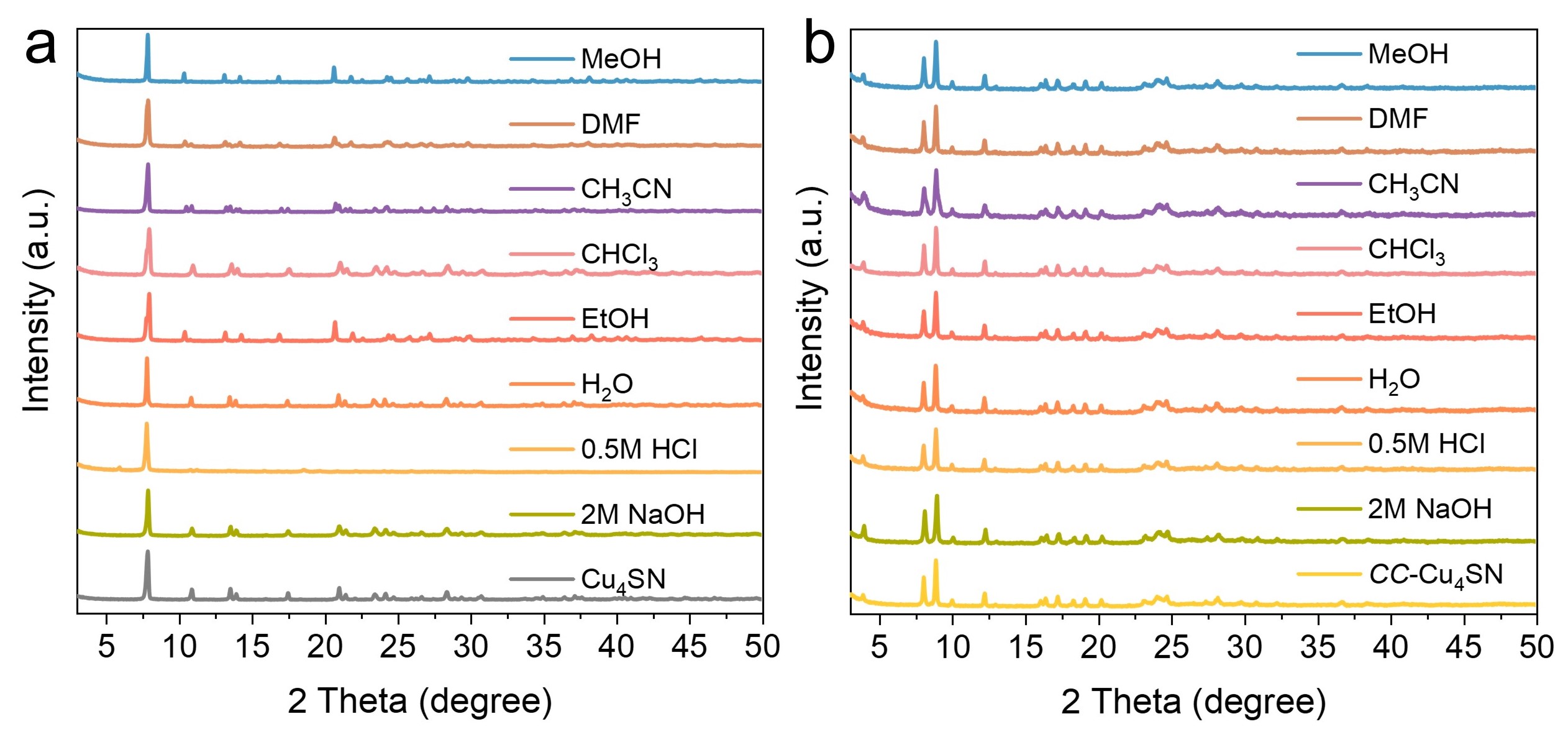


**Figure S16.** PXRD patterns of **Cu_4_SN** (a) and ***CC–*Cu_4_SN** (b) after immersion in various solvents for 48 hours.


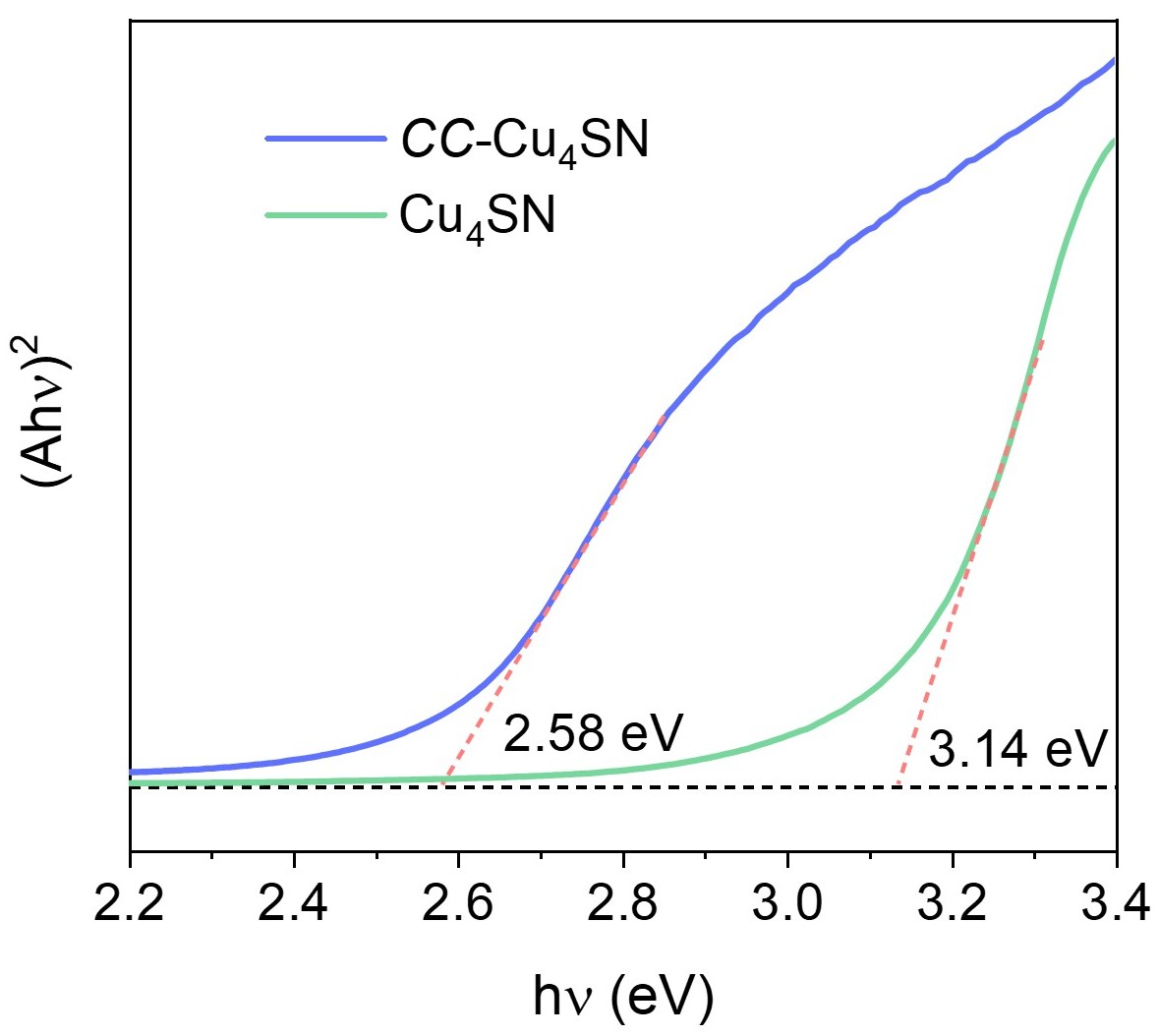


**Figure S17.** Estimated band gaps of **Cu_4_SN** and ***CC–*Cu_4_SN** by Tauc plots.


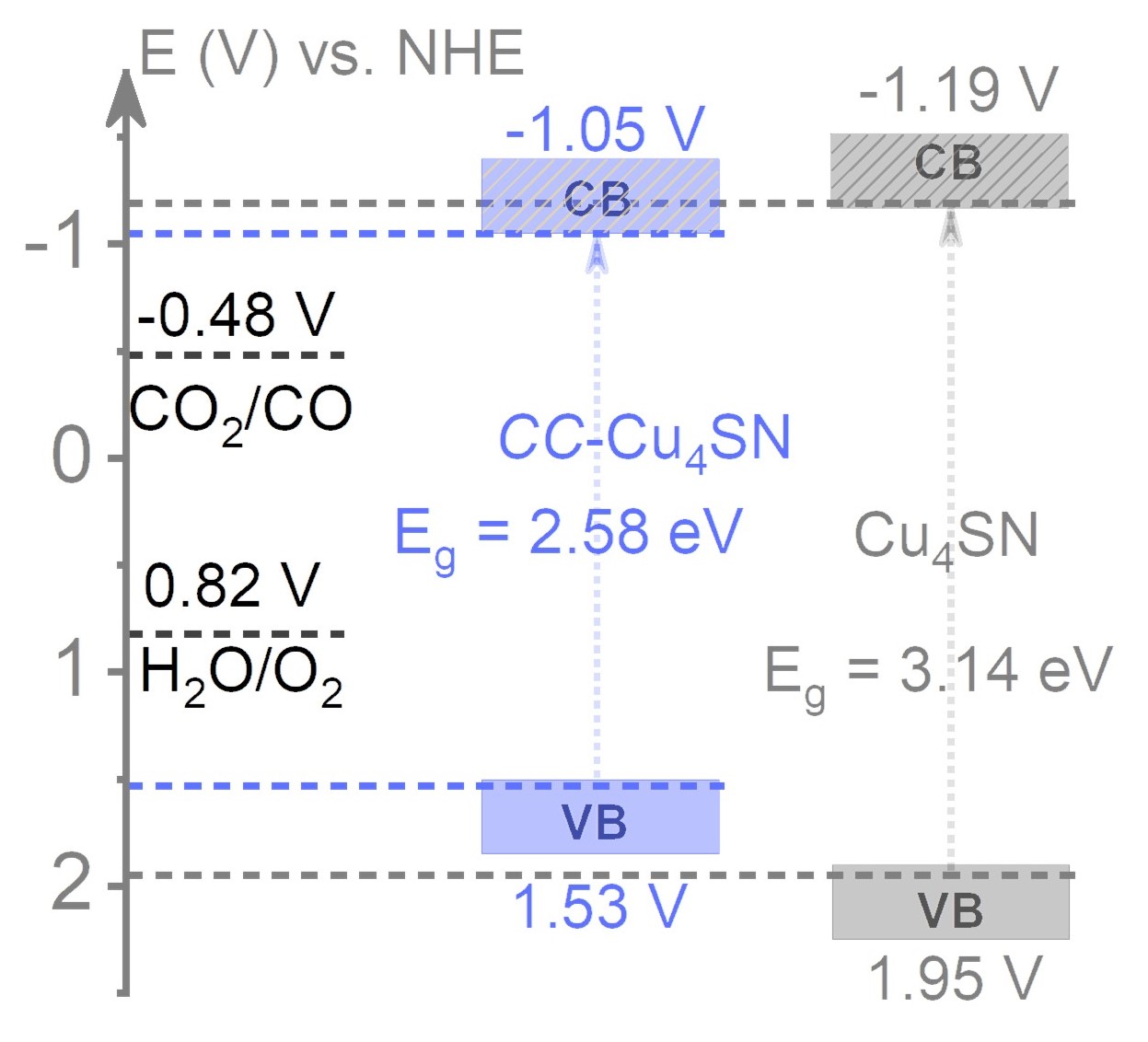


**Figure S18.** Schematic band-structure diagram for **Cu_4_SN** and ***CC–*Cu_4_SN** (CB, conduction band; VB, valence band; NHE, normal hydrogen electrode).


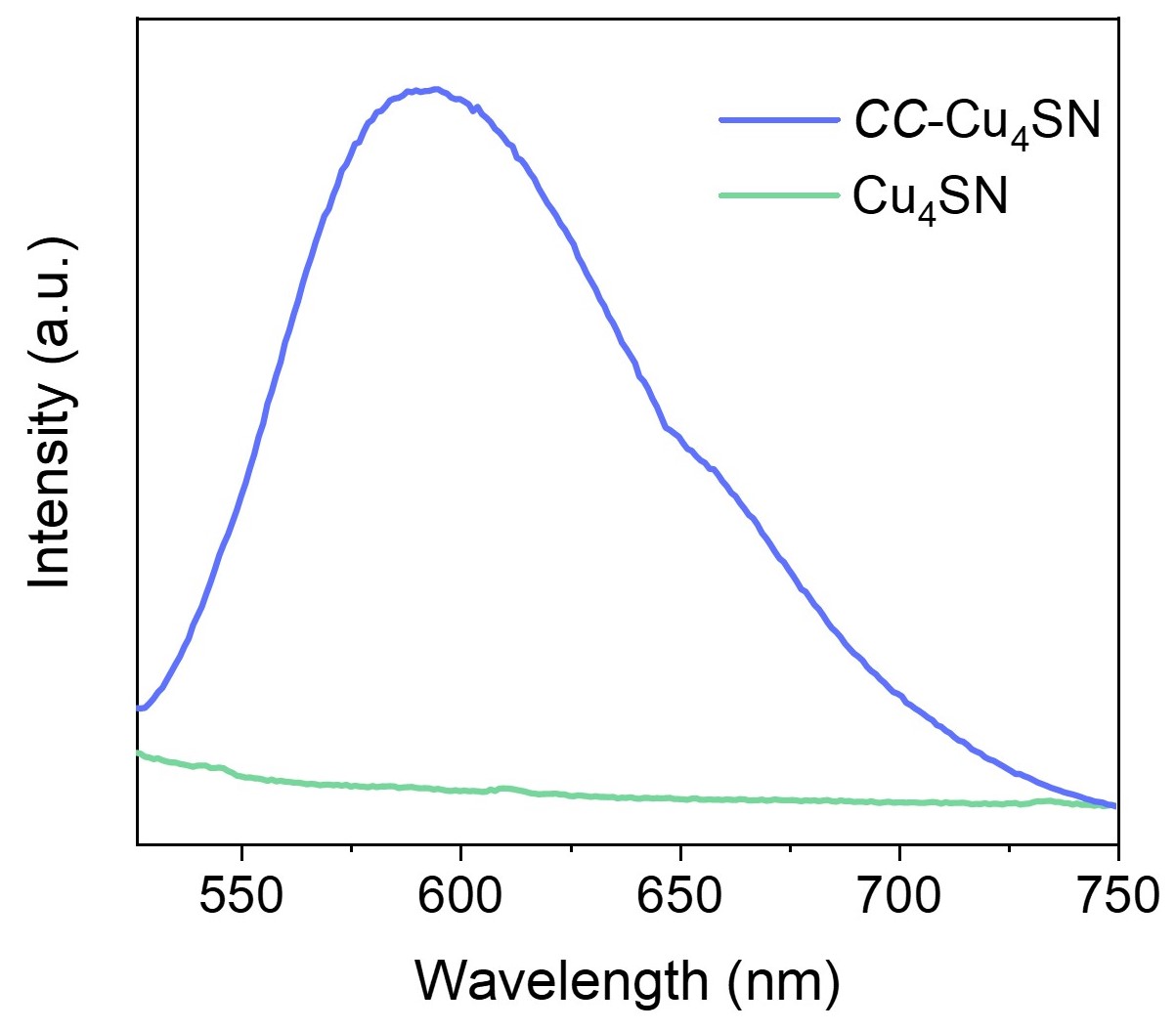


**Figure S19.** PL emissions of **Cu_4_SN** and ***CC–*Cu_4_SN**.


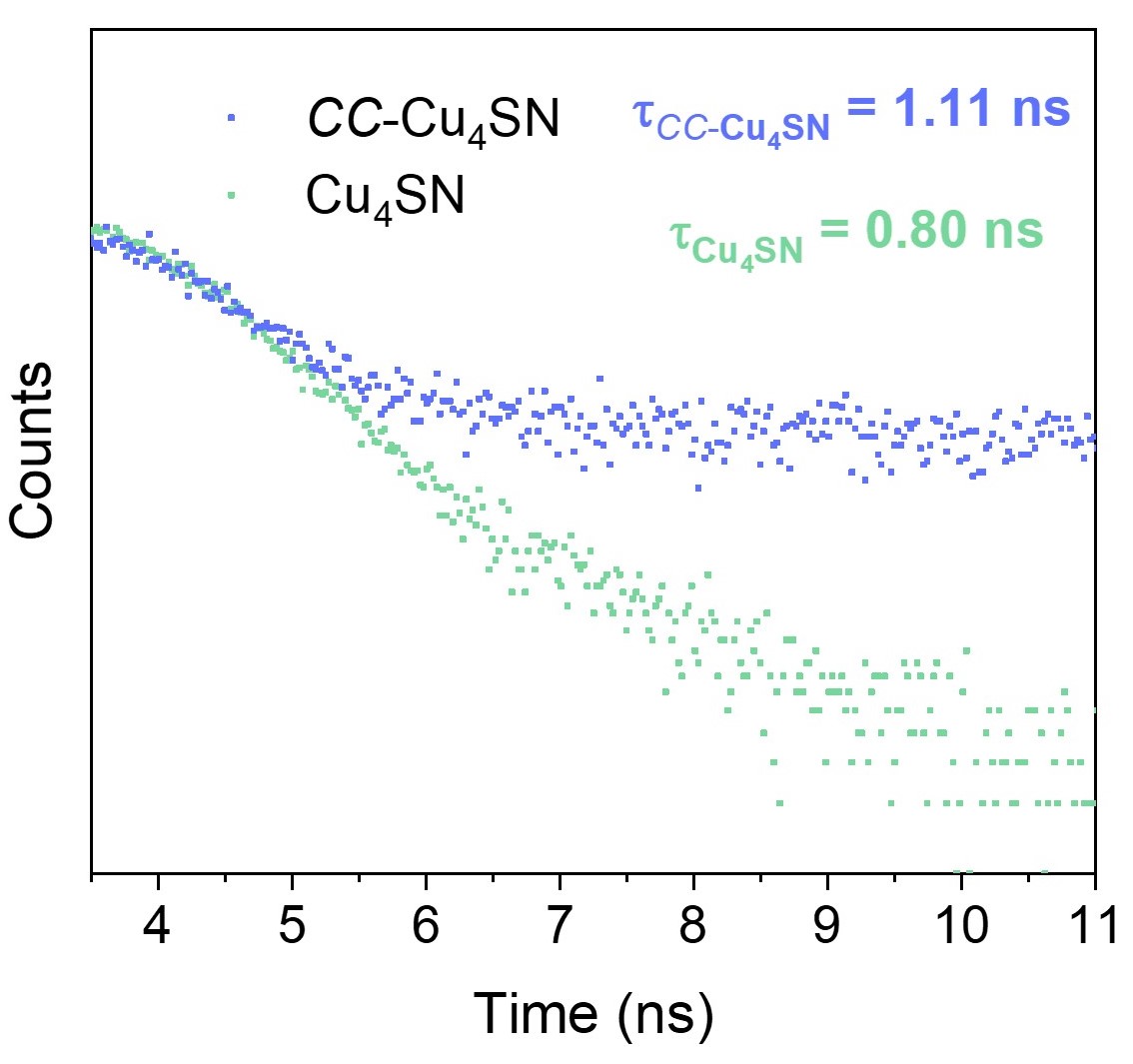


**Figure S20.** Time-resolved PL decay probed at 614 nm for **Cu_4_SN** and ***CC–*Cu_4_SN**.


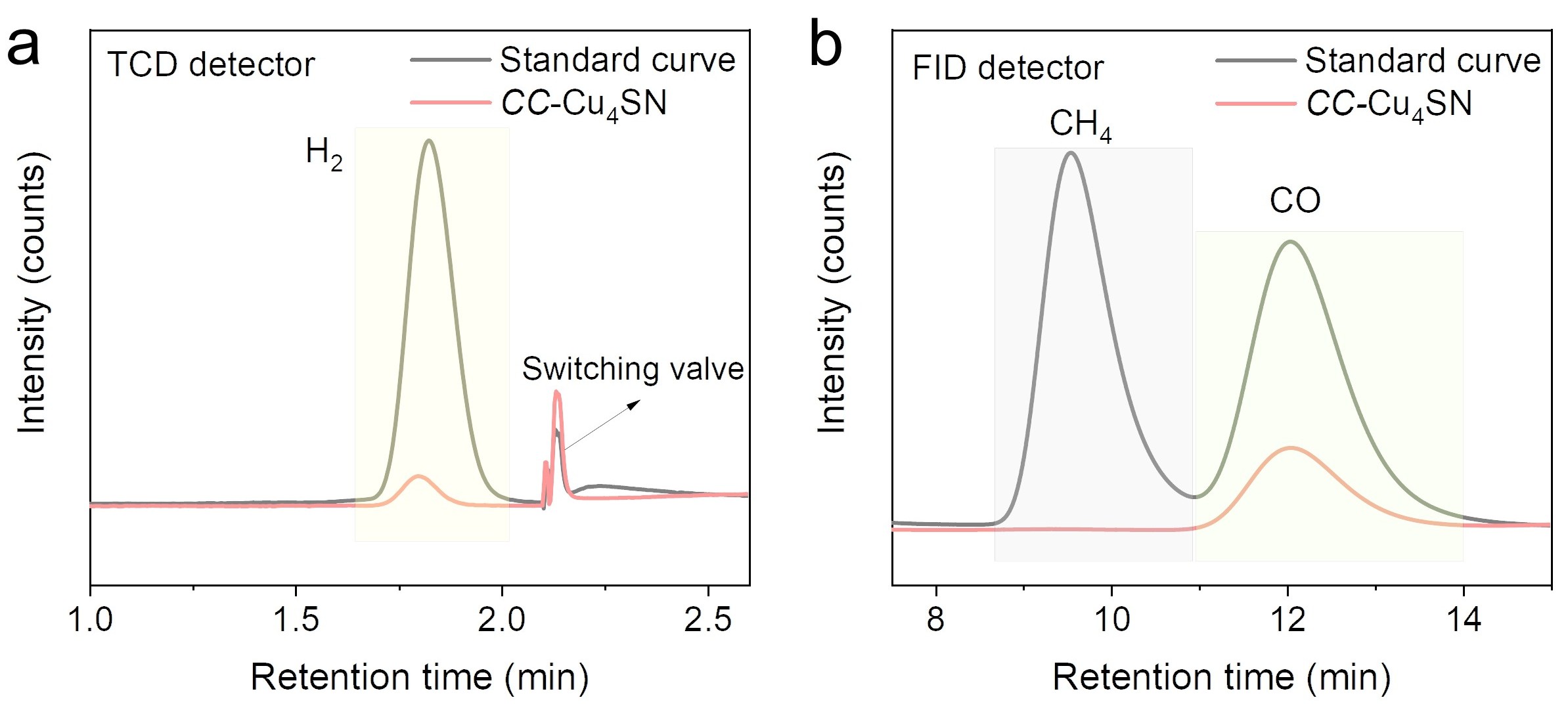


**Figure S21.** Gas chromatograms of products after 6 h of photocatalytic CO_2_ reduction using ***CC*–Cu_4_SN** as the photocatalyst with H_2_O.


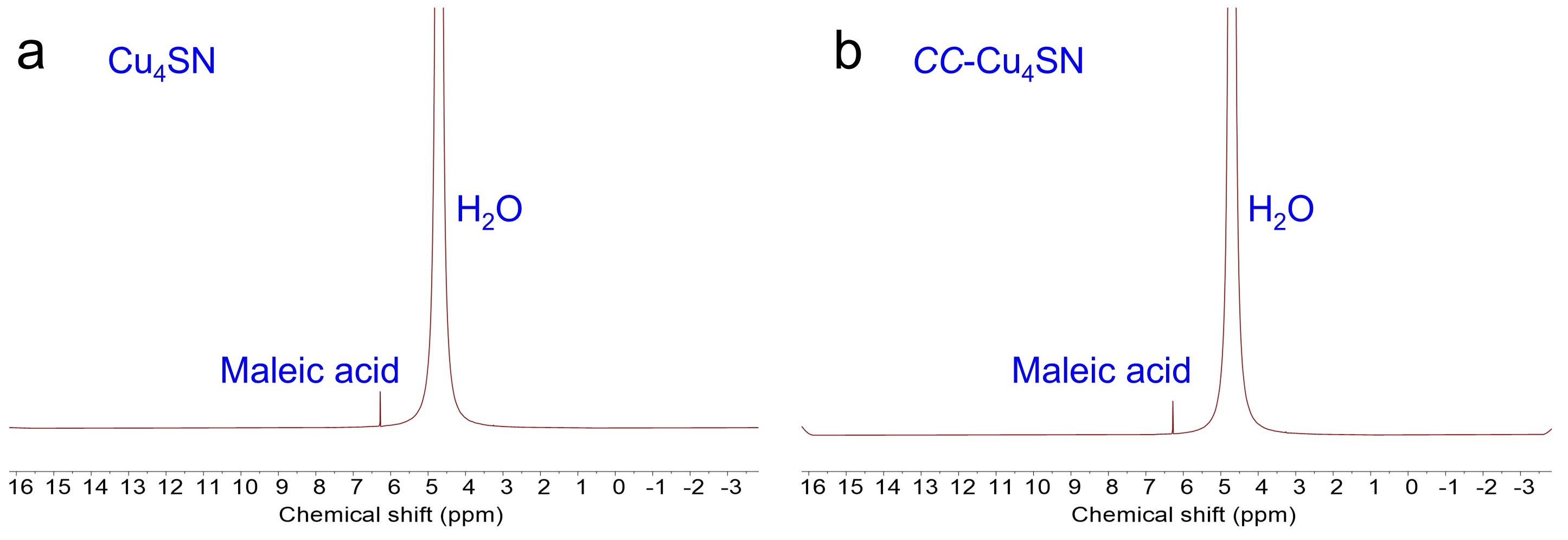


**Figure S22.** ^1^H NMR spectrum of the liquid after photocatalysis for **Cu_4_SN** and ***CC–*Cu_4_SN**. Maleic acid is used as an internal standard.


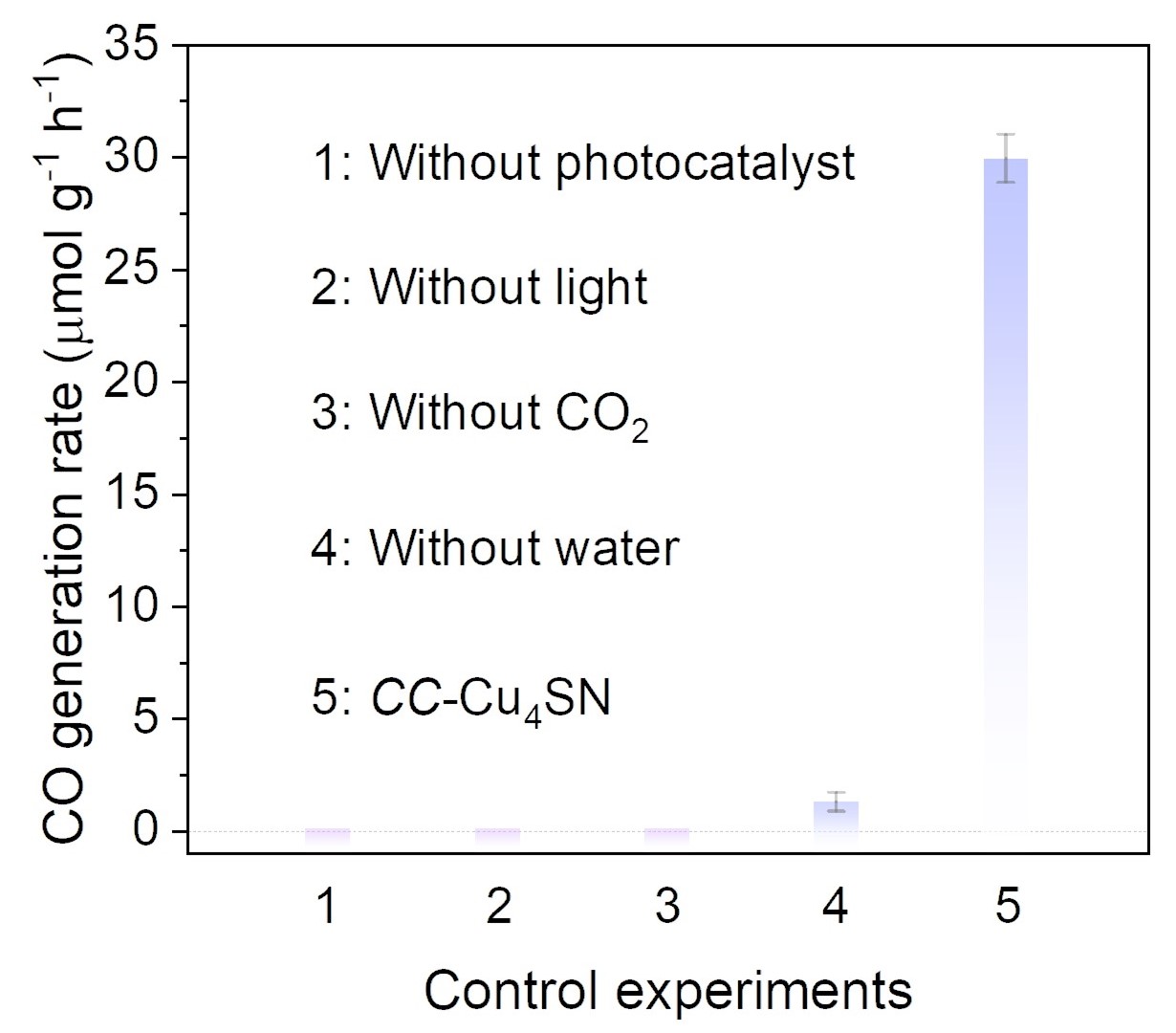


**Figure S23.** Control experiments for ***CC–*Cu_4_SN.**


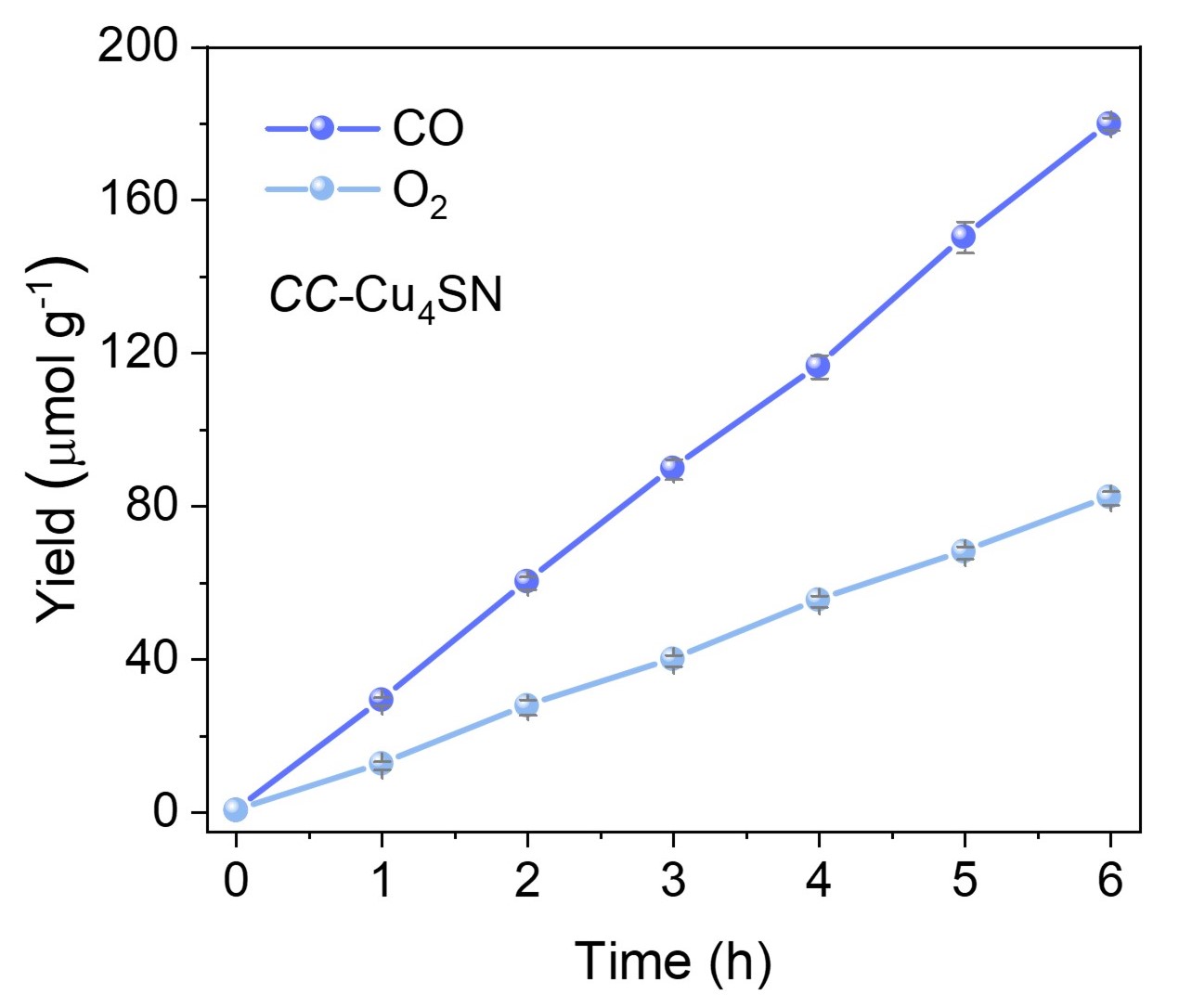


**Figure S24.** CO and O_2_ production during overall CO_2_ photoreduction over ***CC–*Cu_4_SN**.

**
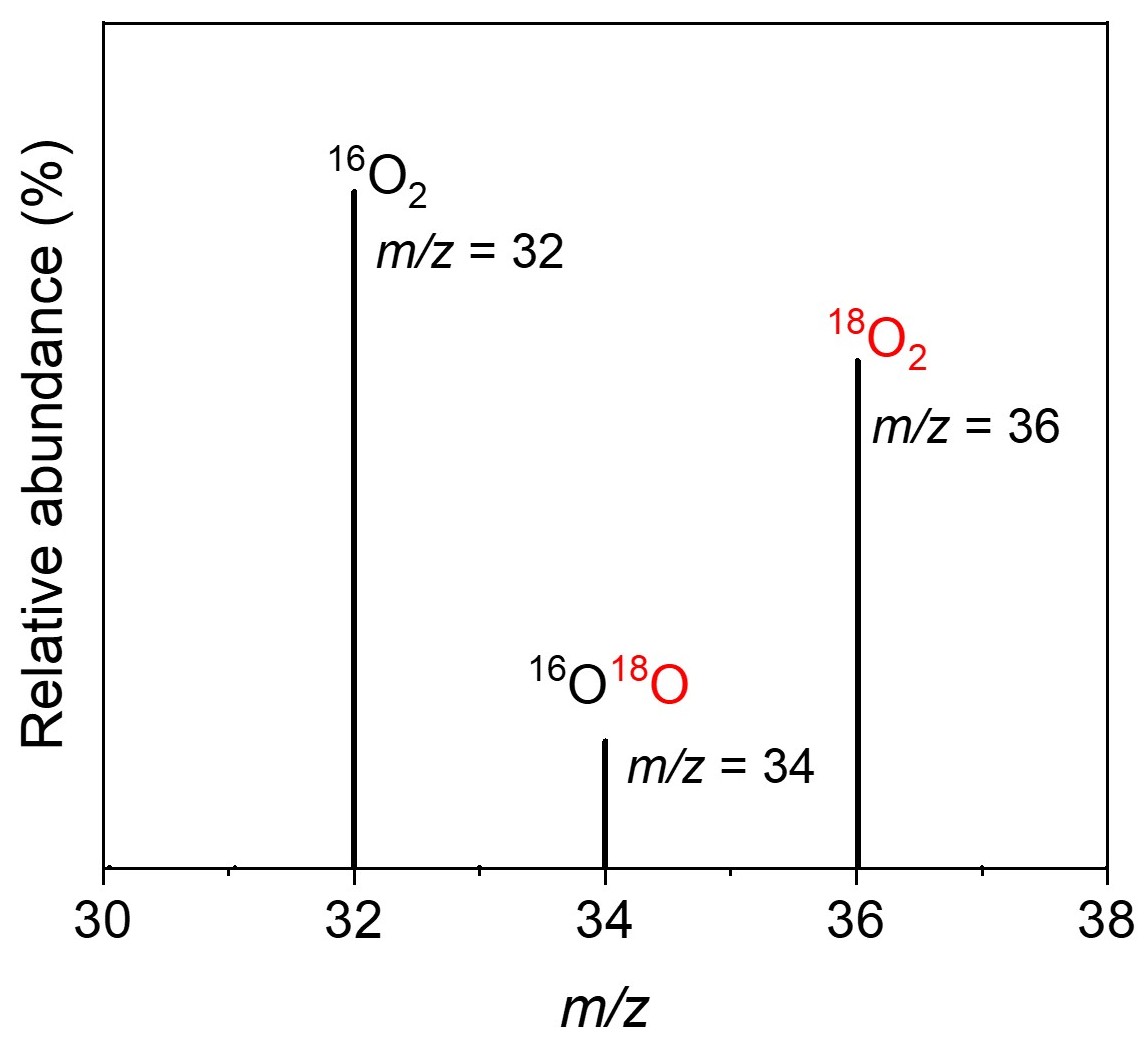
**

**Figure S25.** Mass spectrum (*m/z* =36) analyses of H_2_^18^O labeling experiment over ***CC*–Cu_4_SN**.


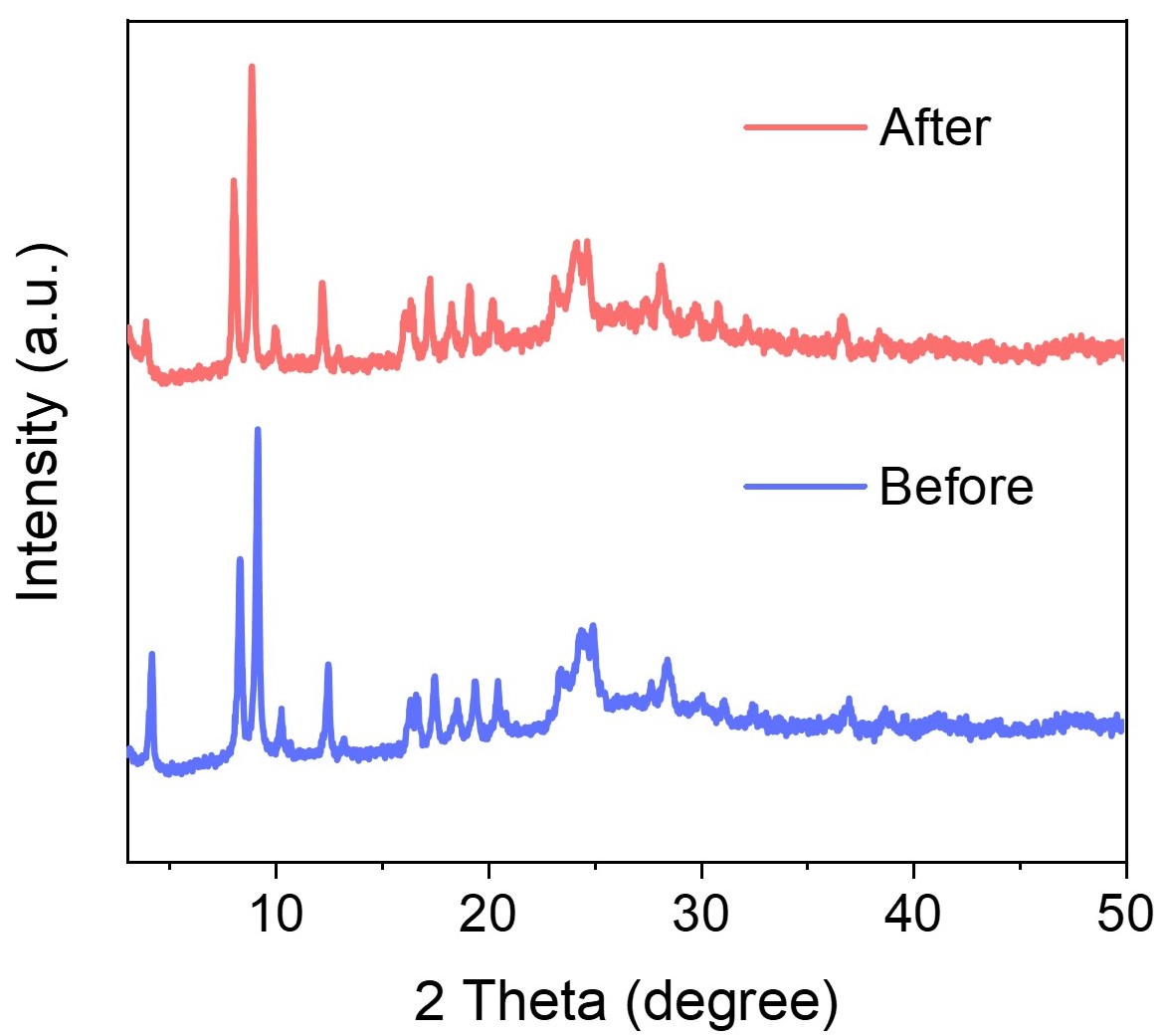


**Figure S26.** PXRD patterns of ***CC–*Cu_4_SN** before and after recycling tests.


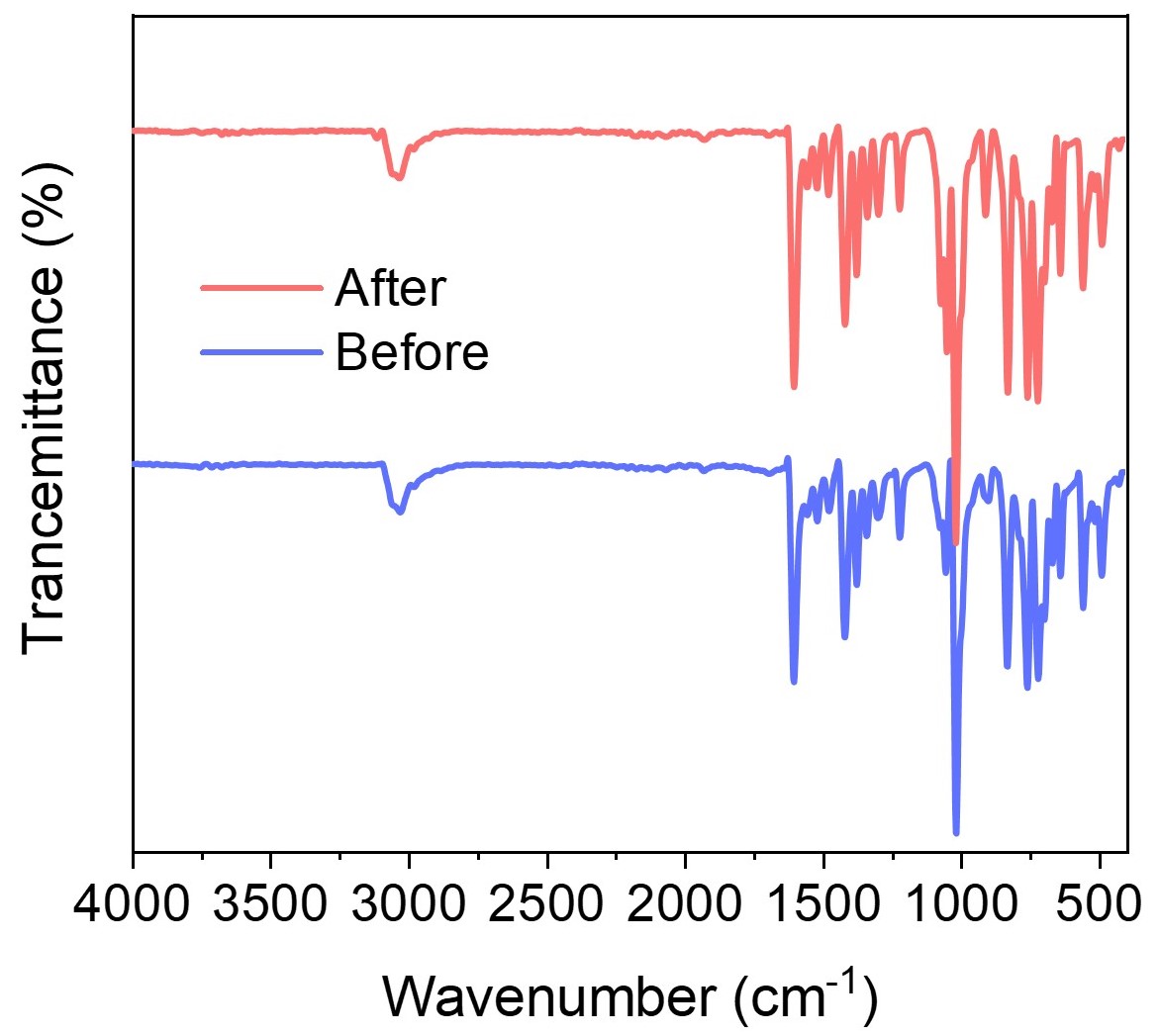


**Figure S27.** FT-IR spectra of ***CC–*Cu_4_SN** before and after recycling tests.


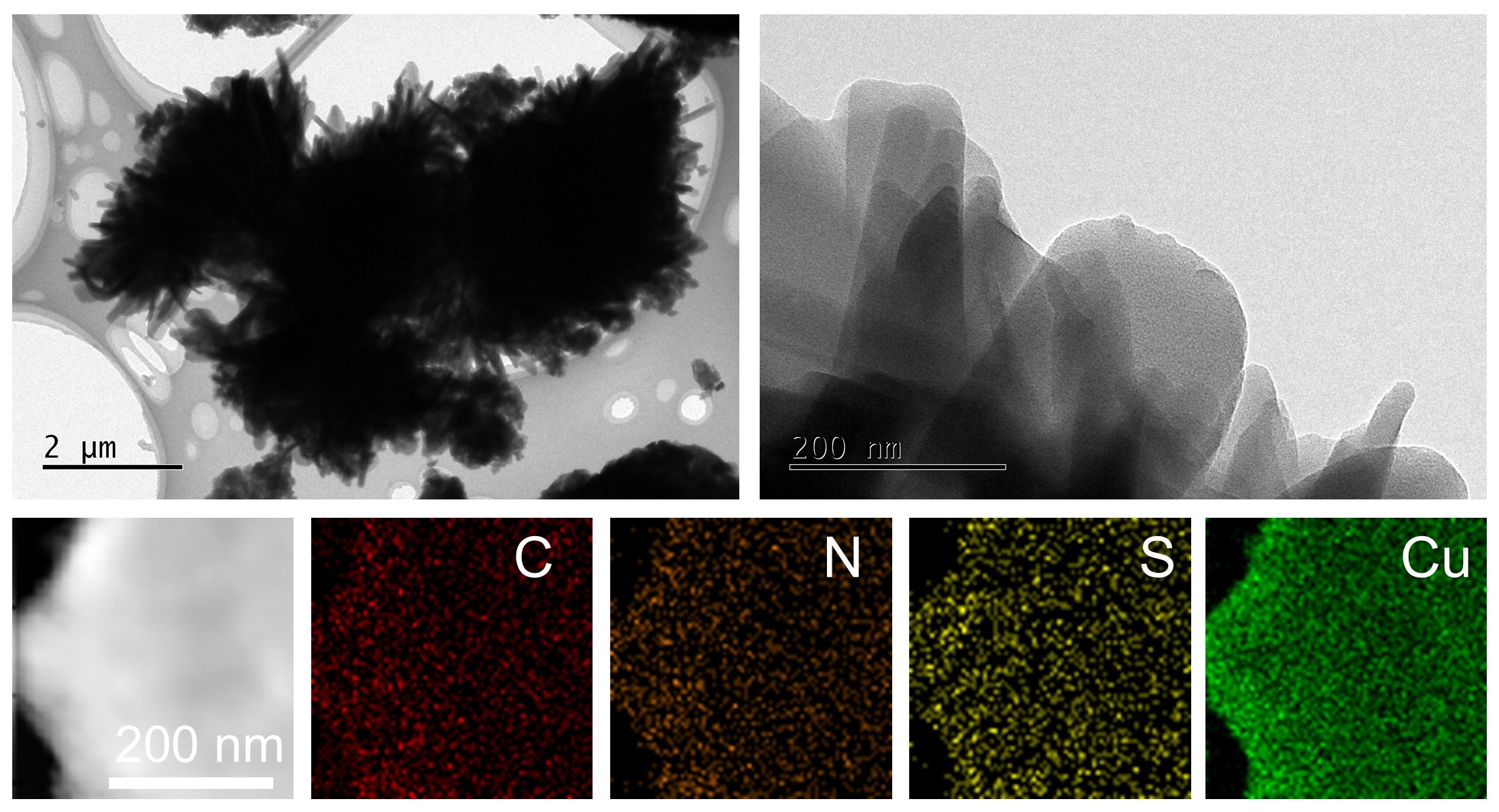


**Figure S28.** HRTEM images and annular-dark-field EDX element mapping of ***CC–*Cu_4_SN** after recycling tests.


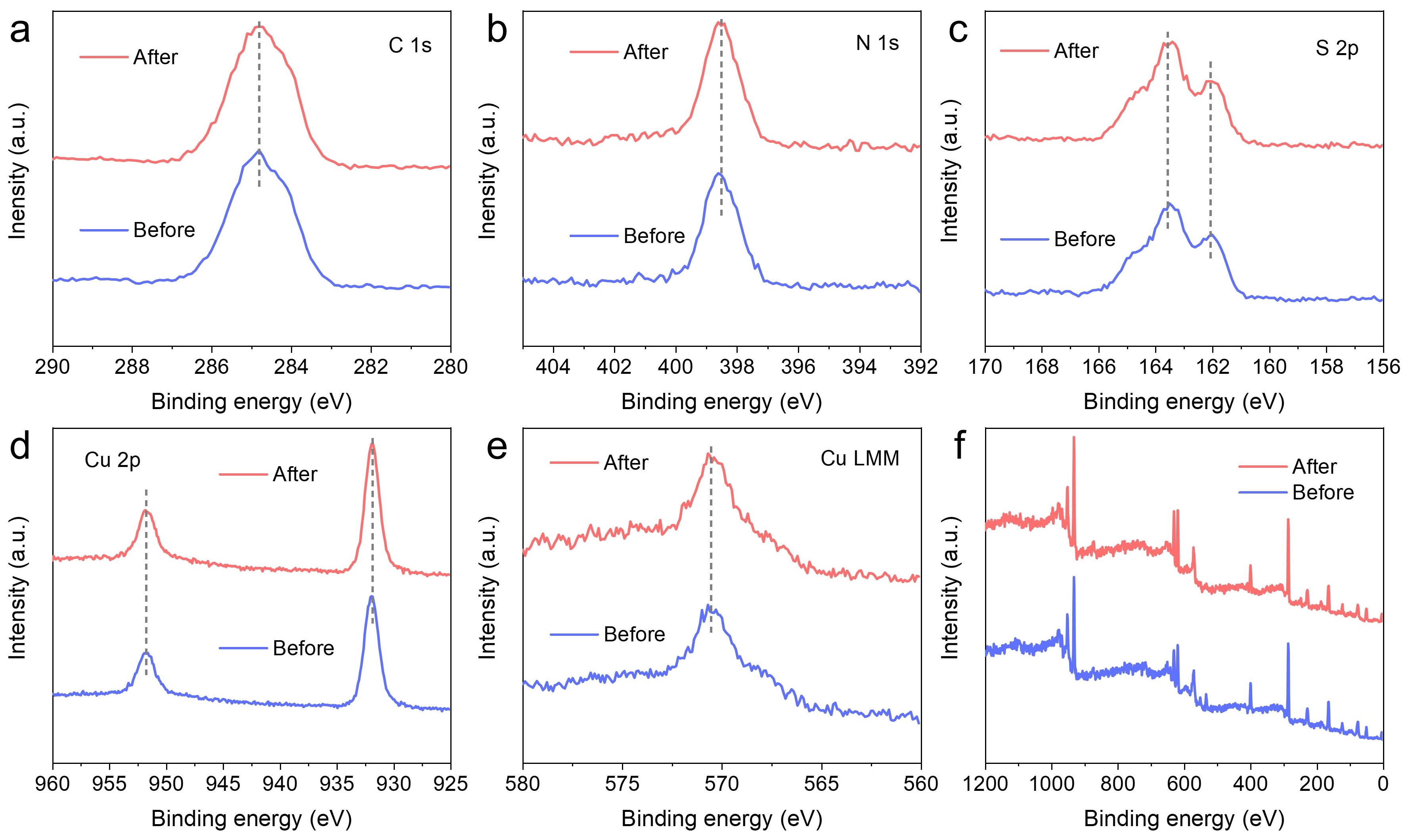


**Figure S29.** XPS spectra of ***CC–*Cu_4_SN** before and after recycling tests.


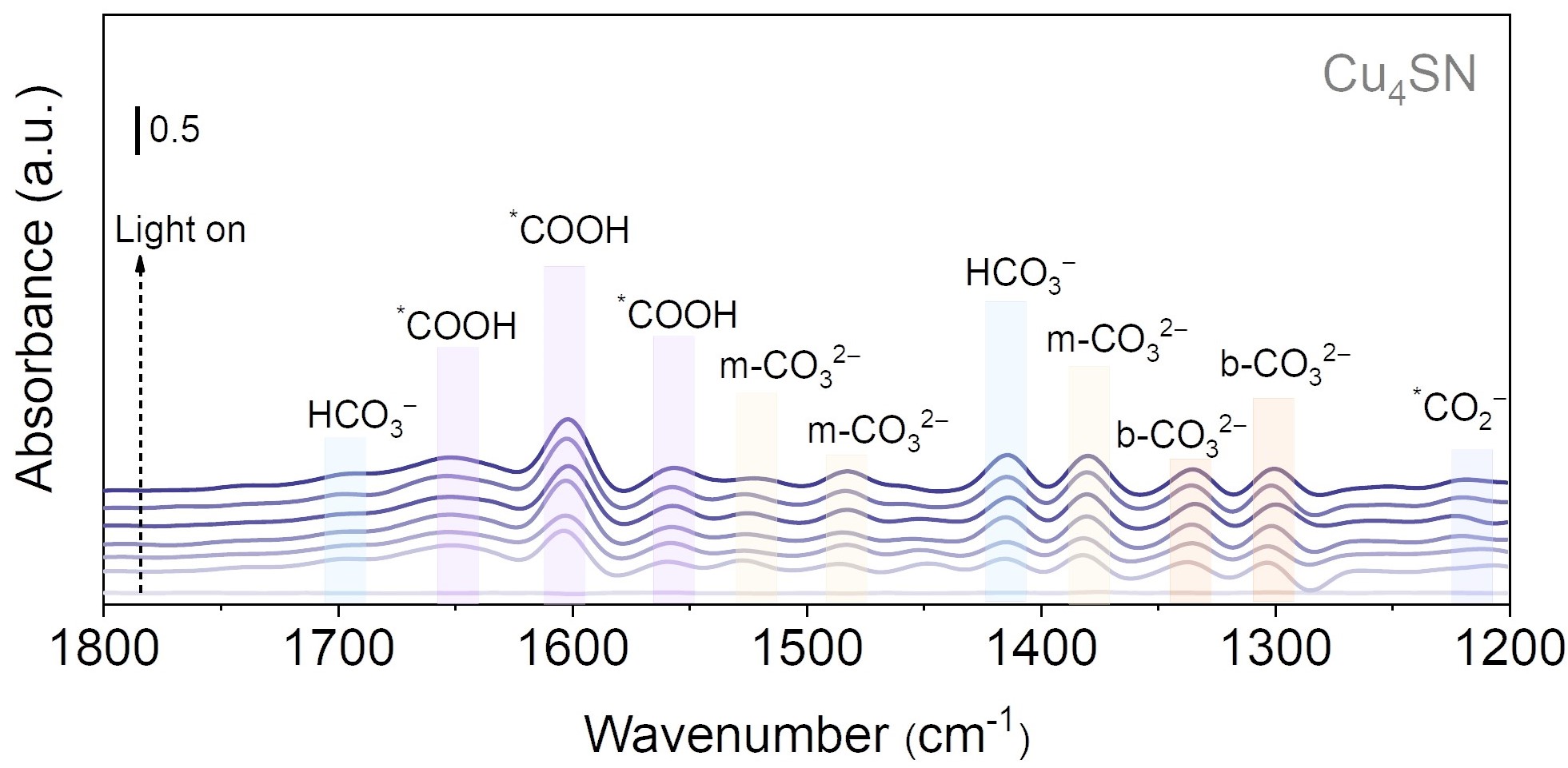


**Figure S30.** In situ FTIR measurements of **Cu_4_SN** under light irradiation.


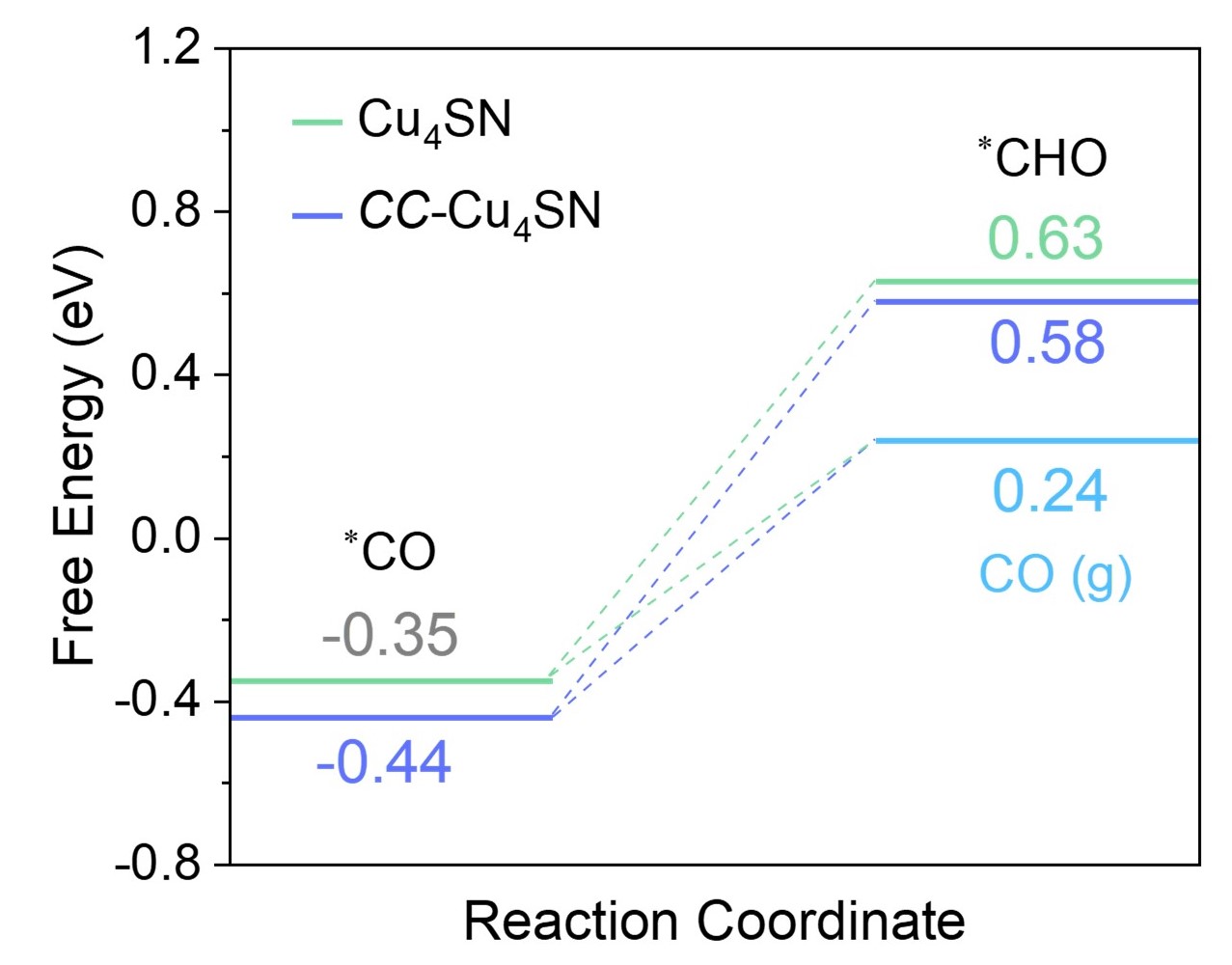


**Figure S31.** Calculated energy barrier for CO desorption and *CHO generation for **Cu_4_SN** and ***CC–*Cu_4_SN**.


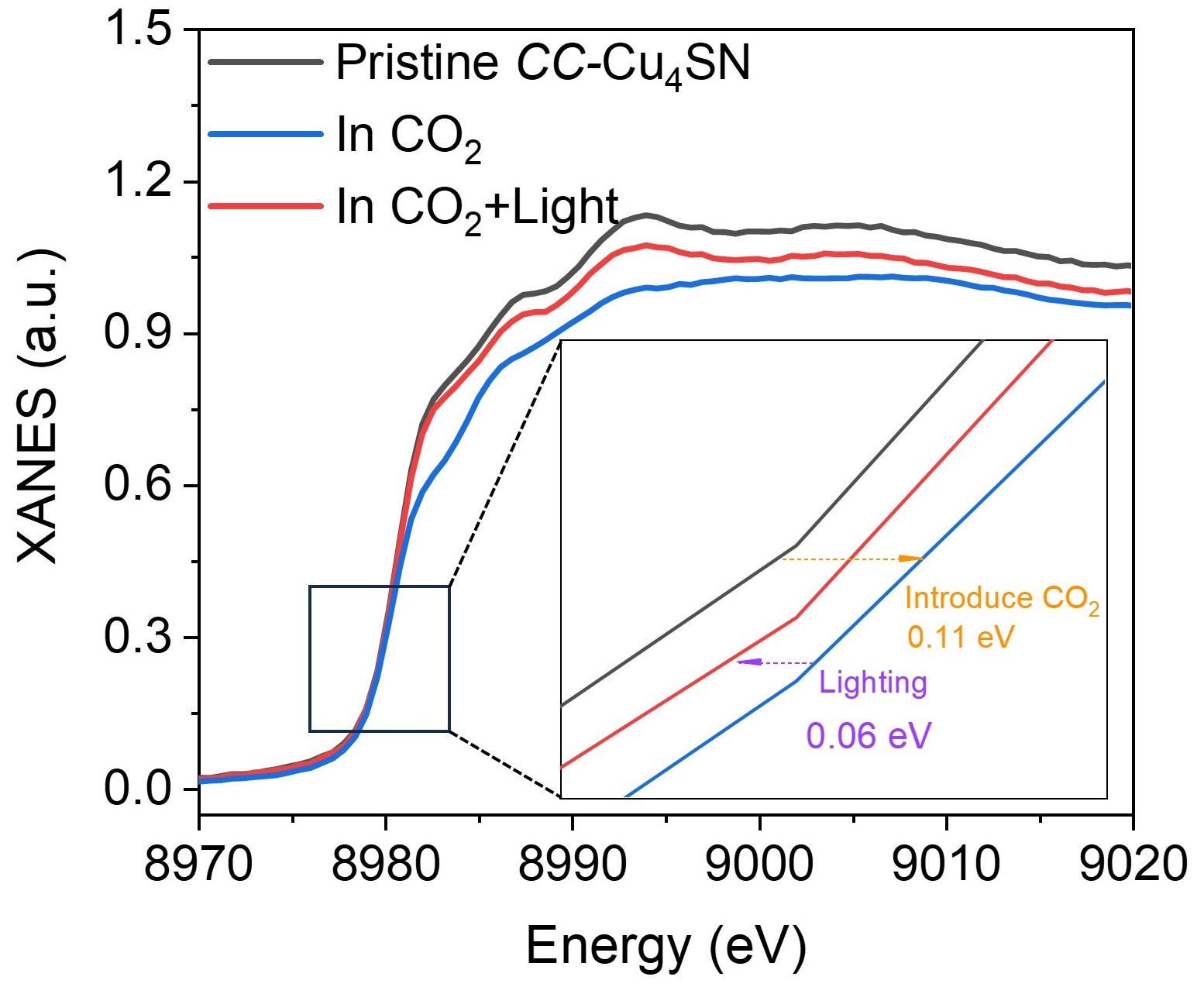
**Figure S32.** Cu K-edge in-situ XANES spectra for ***CC*–Cu_4_SN** in different environments at room temperature.

**Reference**

[1] V. J. A. T. I. *CrysAlisPro*, Santa Clara, CA, USA, **2012**.

[2] G. M. Sheldrick, *Acta Cryst. A* **2015**, *71*, 3-8.

[3] O. V. Dolomanov, L. J. Bourhis, R. J. Gildea, J. A. Howard, H. Puschmann, *J. Appl. Crystallogr.* **2009**, *42*, 339-341.

[4] Diamond - Crystal and Molecular Structure Visualization, Crystal Impact - Dr. H. Putz & Dr. K. Brandenburg GbR, Kreuzherrenstr. 102, 53227 Bonn, Germany, https://www.crystalimpact.de/diamond.

[5] A. Coelho, *J. Appl. Crystallogr.* **2018**, *51*, 210-218.

[6] B. Ravel, M. Newville, *J. Synchrotron Rad.* **2005**, *12*, 537-541.

[7] a) H. Funke, A.C. Scheinost, M. Chukalina, *Phys. Rev. B* **2005***, 71,* 094110; b) H. Funke, M. Chukalina, A. C. Scheinost, *J. Synchrotron Rad.* **2007**, *14*, 426-432.

[8] a) G. Kresse, J. Furthmüller, *Phys. Rev. B* **1996**, *54*, 11169–11186; b) J. Perdew, K. Burke, M. Ernzerhof*, Phys. Rev. Lett.,* **1996**, *77*, 3865–3868; c) G. Kresse, D. Joubert, *Phys. Rev. B* **1999**, *59*, 1758-1775.

[9] a) P. E. Blöchl, *Phys. Rev. B* **1994**, *50*, 17953; b) S. Grimme, J. Antony, S. Ehrlich, H. Krieg, *J. Chem. Phys.* **2010**, *132*, 154104.

[10] G. Henkelman, B. P. Uberuaga, H. Jónsson, *J. Chem. Phys.* **2000**, *113*, 9901-9904.
